# A Facile and General Oxidative Hydroxylation of Organoboron Compounds: Citric Acid as an Efficient Catalyst in Water to Access Phenolic and Alcoholic Motifs

**DOI:** 10.3390/molecules28237915

**Published:** 2023-12-03

**Authors:** Jia-Hui Zhou, Xia Chen, Dan Yang, Chun-Yan Liu, Xiao-Yu Zhou

**Affiliations:** 1 College of Environmental and Chemical Engineering, Dalian University, Dalian 116622, China; zjh17383474825@126.com (J.-H.Z.); cy2403840249@126.com (C.-Y.L.); 2 School of Chemistry and Materials Engineering, Liupanshui Normal University, Liupanshui 553004, China; xia811@live.cn (X.C.); yangda2006@126.com (D.Y.)

**Keywords:** oxidative hydroxylation, organoboron compounds, citric acid, phenols, aliphatic alcohols

## Abstract

An efficient and convenient method for the synthesis of phenols and aliphatic alcohols is described in this paper. The oxidative hydroxylation reaction of various organoboron compounds proceeded smoothly by employing H_2_O_2_ as the oxidant and citric acid as the catalyst in water at room temperature to produce phenols and aliphatic alcohols in satisfactory to excellent yields (up to 99% yield). Various synthetically useful functional groups, such as halogen atom, cyano, and nitro groups, remain intact during the oxidative hydroxylation. The developed catalytic system also could accommodate phenylboronic pinacol ester and potassium phenyltrifluoroborate to give the target product good yields.

## 1. Introduction

Phenols and aliphatic alcohols are widely recognized as privileged building blocks for the construction of sophisticated organic complexes such as polymers, pharmaceuticals, and natural products [1,2,3]. Accordingly, the development of efficient and convenient methods for preparation has attracted considerable attention. Because of the unique properties of boronic acids, such as ready availability and stability in air and aqueous environments, the oxidative hydroxylation of boronic acids has become a straightforward and efficient avenue to access phenols and aliphatic alcohols. This process relies on the use of molecular oxygen (O_2_ and air) [4,5,6,7,8,9,10] and stoichiometric oxidants such as hydrogen peroxide (H_2_O_2_) [11,12,13,14,15,16,17,18], *t*-butylhydroperoxide (TBHP) [19], *m*-chloroperbenzoic acid (*m*CPBA) [20], hydroxylamine [21], *N*-oxides [22], hypervalent iodine [23], and sodium chlorite (NaClO_2_) [24]. In view of both the economy and the environment, molecular oxygen and H_2_O_2_ are the ideal oxidants due to their low cost and lack of toxic byproducts. However, virtually all of the methods for the use of molecular oxygen were achieved using metal catalysts and relatively long reaction times. In general, metal catalysts are expensive, air or moisture sensitive, and can be a challenging task to remove; thus, from the practical viewpoint, metal-free processes are much more preferred in the pharmaceutical industry [14,22]. Therefore, more benign, cost-effective, environmentally friendly metal-free procedures have emerged as beneficial alternatives.

Several metal-free procedures using H_2_O_2_ as an oxidant have been developed for the synthesis of phenols in various reaction systems, such as aqueous H_2_O_2_ [11], H_2_O_2_-I_2_ [25], H_2_O_2_-poly(*N*-vinylpyrrolidone) [26], H_2_O_2_-amberlite IR-120 resin [27], H_2_O_2_-PEG400 [28], H_2_O_2_-silica chloride [29], H_2_O_2_-ascorbic acid [14], H_2_O_2_-graphene oxide [30], and H_2_O_2_-boron nitride nanosheets [31]. These protocols often show high efficiency; however, most of the methodologies involve organic solvents [25,26,28,29], a large amount of H_2_O_2_ [14,25,27], complex reagents [26,30,31], and heating conditions [31], which are difficult to practice in industrial applications. Furthermore, in many of these methods, both the scaled-up synthesis of phenols and the compatibility with alkylboronic acids have not been demonstrated. Thus, a general, convenient, and eco-friendly protocol is still in demand.

Citric acid, the principal organic acid found in citrus fruits, is a nontoxic, low cost, biodegradable, and environmentally benign catalyst in organic transformations [32,33,34,35]. Green chemistry that aims to achieve transformations with the avoidance of using and generating hazardous substances and minimize energy consumption is highly sought after in modern organic synthesis [36]. As such, the use of environmentally non-compatible catalysts and organic solvents should be avoided to do away with detrimental effects such as toxicity, hazards, and waste treatment. Notably, using a natural substance as a catalyst and water (H_2_O) as a solvent at room temperature offers various benefits to address these problems. Herein, we are committed to developing a convenient, green, and general method for the synthesis of phenols and aliphatic alcohols via the oxidative hydroxylation of organoboron compounds occurring in the presence of citric acid under mild conditions.

## 2. Results

As a model reaction, we first investigated citric acid-catalyzed oxidative hydroxylation of phenylboronic acid (**1a**, 1 equiv.) under various conditions (Table 1). Given the fact that citric acid has good water solubility and boronic acids have high stability in aqueous environments, we reasoned that the oxidative hydroxylation of boronic acids to access phenolic and alcoholic motifs could be conducted in H_2_O instead of the organic solvents. Therefore, the reaction temperature was first screened using H_2_O_2_ as the oxidant and H_2_O as the solvent. The results obtained indicated that room temperature with no extra energy consumption was the best reaction temperature (entry 3 vs. entries 1 and 2). The oxidant was subsequently investigated under room temperature conditions. Among the examined oxidants (H_2_O_2_, air, TPHP, di-*t*-butyl peroxide (DTBP), O_2_, sodium chlorite (NaClO_2_)), H_2_O_2_ proved to be the best oxidant, providing the target product, phenol (**3a**), in 98% yield (entry 3 vs. entries 4–8). Decreased yields were observed when the loadings of oxidant H_2_O_2_ and catalyst citric acid were reduced to 1.0 equivalent and 0.05 mol%, respectively (entries 9 and 11 vs. entry 3); on the other hand, quantitative yield was almost obtained even though the loadings of oxidant H_2_O_2_ and catalyst citric acid increased to 3.0 equivalents and 0.20 mol%, respectively (entries 10 and 12 vs. entry 3). The results suggested that the target reaction was commendably accomplished using 2.0 equivalents of oxidant H_2_O_2_ and 0.10 mol% of catalyst citric acid. Decreased yield was also obtained when the reaction time was shortened to 1 h from 2 h (entry 13 vs. entry 3). Moreover, the oxidative hydroxylation reaction of **1a** was performed without citric acid and only provided **2a** in 66% yield, suggesting that the presence of citric acid in the reaction system influenced the conversion efficiency significantly (entry 14 vs. entry 3). The influence of acid catalysts on oxidative hydroxylation was also investigated. Acetic acid and benzoic acid were not able to significantly improve the yield (entries 15 and 16 vs. entry 14), while tartaric acid and formic acid had some positive effect on this reaction, although it was not as good as citric acid (entries 17 and 18 vs. entry 3). Therefore, the subsequent oxidative hydroxylation reaction of various boronic acids with H_2_O_2_ (2.0 equiv.) was performed in the presence of citric acid (0.1 mol%) in H_2_O (3 mL) at room temperature.

**Scheme 1:**
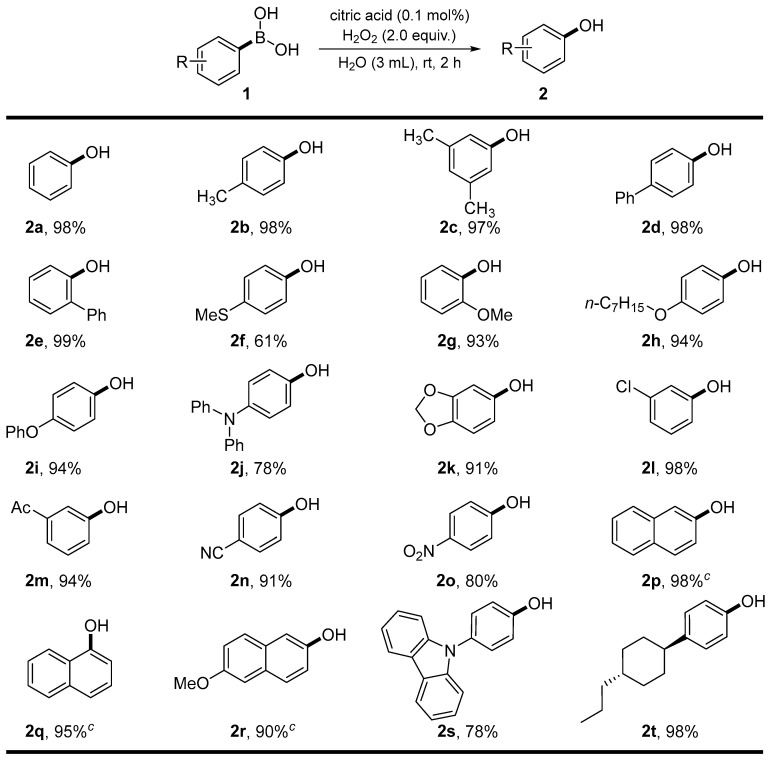
Substrate scope ^a,b^. ^a^ Reaction conditions: **1** (1.0 mmol), H_2_O_2_ (2.0 mmol, 2.0 equiv.), and citric acid (0.2 mg, 0.001 mmol, 0.1 mol%) in H_2_O (3 mL) at room temperature under an air atmosphere for 2 h. ^b^ Isolated yield. ^c^ The reaction was performed for 4 h.

Given the optimized reaction conditions, the scope and limitation of this reaction were explored, and the results are summarized in Scheme 1. The methyl (Me), cyclohexyl, and phenyl (Ph) linked on different positions of the benzene ring in substrate **1** did not influence the yield; the corresponding phenol products **2b**–**2e** and **2t** were obtained in 97–99% yields. Other electron-donating groups, such as methylthio (MeS), alkoxyl (MeO and *n*-C_7_H_15_O), phenoxyl (PhO), and diphenylamino (Ph_2_N), were also tolerated well in this oxidative hydroxylation reaction, providing the desired products **2f**–**2j** in 61–94% yields, respectively. Reactions of boronic acid substrates **1** bearing electron-withdrawing group on the benzene ring, such as acetyl (Ac), cyano (CN), and nitro (NO_2_), were subsequently investigated under the optimized reaction conditions. The phenol products **2m**–**2o** were obtained in good to excellent yields (80–94%). These results mentioned above indicated that the electron properties (electron donating or electron withdrawing) of substituents linked to the benzene ring did not influence the reactivities of **1**. Notably, phenylboronic acid bearing a chloro substituent was successfully transformed to 3-chlorophenol (**2l**) in 98% yield. The functional groups, such as Cl, CN, and NO_2_, remained intact during the oxidative hydroxylation reaction, suggesting that further manipulation may produce additional useful compounds. The suitability of naphthalene-derived boronic acids **1p**–**1r** in the current oxidative hydroxylation reaction was investigated, and excellent yields (91–98%) of the desired products **2p**–**2r** were obtained, respectively. The desired products **2k** and **2s** were also obtained in 91% and 78% yields, respectively, from the reactions of benzo[*d*][1,3]dioxol-5-ylboronic acid (**1k**) and (4-(9*H*-carbazol-9-yl)phenyl)boronic acid (**1s**). In general, this oxidative hydroxylation exhibits broad scope and proceeds efficiently with electron-poor and -rich arylboronic acids, even with significant steric hindrance.

**Scheme 2:**
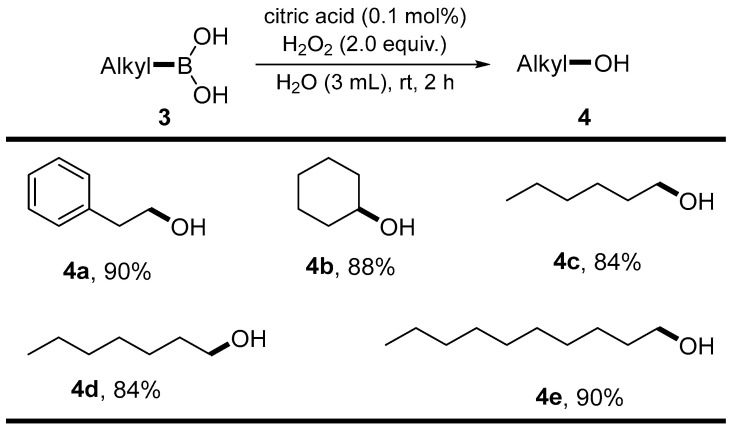
Substrate scope ^a,b^. ^a^ Reaction conditions: **1** (1.0 mmol), H_2_O_2_ (2.0 mmol, 2.0 equiv.), and citric acid (0.2 mg, 0.001 mmol, 0.1 mol%) in H_2_O (3 mL) at room temperature under an air atmosphere for 2 h. ^b^ Isolated yield.

Encouraged by these results, we next explored the scope of this reaction with respect to alkylboronic acids. As shown in Scheme 2, various alkylboronic acids **3a**–**3e** were smoothly converted into the desired products **4a**–**4e** in good to excellent yields (84–90%). Notably, aliphatic alcohols are generally susceptible to oxidation conditions and could be readily transformed into the corresponding aldehydes or ketones. Advantageously, alcohols are able to survive under our organocatalytic oxidation conditions.

**Scheme 3:**
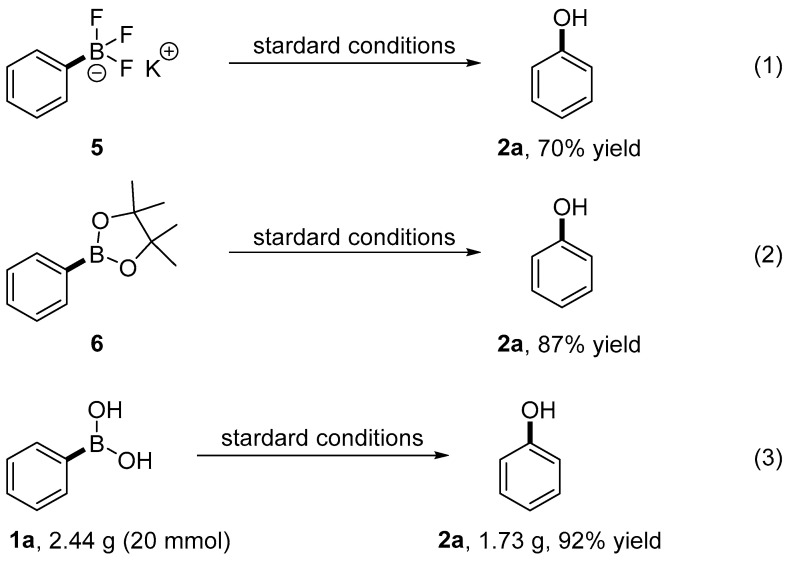
Substrate extension studies and large-scale synthesis.

Continuous substrate extension studies showed that the potassium phenyltrifluoroborate (**5**) and phenylboronic pinacol ester (**6**) could be used as a phenyl source to synthesize phenol under the standard conditions (Scheme 3, (1) and (2)). Large-scale synthesis of phenol (**2a**) was performed under optimal reaction conditions to demonstrate the synthetic practicality and utilization of the new methodology further (Scheme 3, (3)). As expected, the desired products **2a** were obtained in excellent yield when the reaction of **1a** was conducted on a 20.0 mmol scale. The yield is comparable to that obtained in the small-scale experiment.

**Scheme 4:**
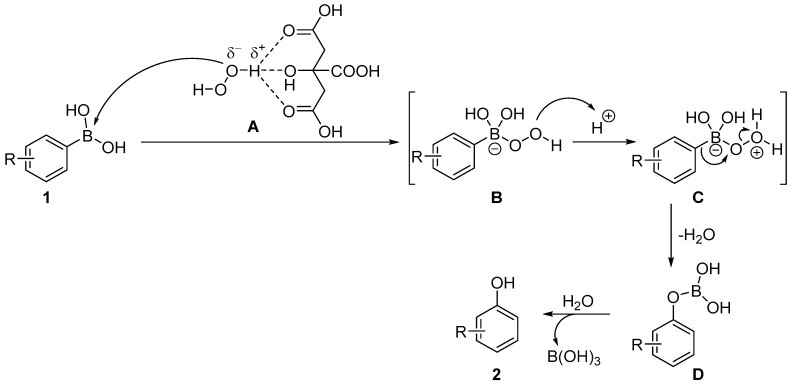
The plausible reaction mechanism.

Based on the experimental results and previous reports [11,12,13,14,28,29], a plausible reaction mechanism for the current oxidative hydroxylation is depicted in Scheme 4. Initially, the citric acid-activated H_2_O_2_ species **A** was generated in situ through a hydrogen bond. The activated H_2_O_2_ species then underwent nucleophilic attack on boronic acid **1** to form a key intermediate, boron peroxo complex **B**, followed by phenyl migration in the form of **C** to a peroxygen atom to generate boron phenoxide **D**. Finally, the hydrolysis of **D** occurred to yield the target product **2**.

## 3. Materials and Methods

### 3.1. General Information

Unless otherwise noted, all reactions were carried out in oven-dried 25 mL Schlenk tubes under a nitrogen atmosphere. An IKA plate was used as the heat source. All reagents and solvents were of pure analytical grade. Thin-layer chromatography (TLC) was performed on HSGF254 silica gel, pre-coated on glass-backed plates coated with 0.2 mm silica and revealed with a UV lamp (λ_max_ = 254 nm). The products were purified by flash column chromatography on silica gel 200–300 mesh. ^1^H and ^13^C NMR spectra were recorded on a Varian Inova-500 spectrometer (500 MHz for ^1^H, 126 MHz for ^13^C) and a Bruker Avance NEO 600M NMR Spectrometer (600 MHz for ^1^H, 151 MHz for ^13^C) using CDCl_3_ as the solvent with tetramethylsilane (TMS) as the internal standard at room temperature. The chemical shifts are reported in ppm downfield (*δ*) from TMS, and the coupling constants *J* are given in Hz. The peak patterns are indicated as follows: s, singlet; d, doublet; dd, doublet of doublet; t, triplet; q, quartet; p, quintet; h, sextet; m, multiplet (see Supplementary Materials). High-resolution mass spectra were recorded on either a Q-TOF mass spectrometer or an LTQ Orbitrap XL mass spectrometer. 

### 3.2. Synthetic Procedures

#### 3.2.1. The Typical Procedure for the Oxidative Hydroxylation of Arylboronic Acids

A mixture of arylboronic acids **1** (1.0 mmol) and citric acid (0.2 mg, 0.001 mmol, 0.1 mol%) in H_2_O (3 mL) was added into a Schlenk flask (25 mL). Then, 30% aqueous H_2_O_2_ (227 µL, 2.0 eq.) was added and stirred at room temperature under an air atmosphere for 2 h. After the reaction was finished, the reaction mixture was diluted with water and then extracted with ethyl acetate. The combined organic layer was dried on sodium sulfate and filtered. Then, the solvent was evaporated under reduced pressure and the residue was purified by column chromatography (petroleum ether/ethyl acetate 5:1 to 2:1) to provide the product **2**.

*phenol* (**2a**): Yield: 98%, 92.2 mg, light pink solid, mp 38–40 °C, R_f_ = 0.50 (H/E = 5:1). ^1^H NMR (500 MHz, CDCl_3_) *δ* 7.29 (d, *J* = 6.2 Hz, 2H), 6.97 (t, *J* = 7.4 Hz, 1H), 6.87 (d, *J* = 7.7 Hz, 2H), and 4.64 (brs, 1H). ^13^C NMR (126 MHz, CDCl_3_) *δ* 155.5, 129.7 (2C), 120.8, and 115.3 (2C). [M + H]^+^ calcd for C_6_H_7_O, 95.0497; found 95.0493.
*p-cresol* (**2b**)**:** Yield: 98%, 105.7 mg, light brown solid, mp 30–32 °C, R_f_ = 0.52 (H/E = 5:1). ^1^H NMR (500 MHz, CDCl_3_) *δ* 7.06 (d, *J* = 8.0 Hz, 2H), 6.76 (d, *J* = 8.2 Hz, 2H), and 4.72 (brs, 1H), 2.30 (s, 3H). ^13^C NMR (126 MHz, CDCl_3_) δ 153.2, 131.0, 130.1 (2C), and 115.1 (2C), 20.5. [M + H]^+^ calcd for C_7_H_9_O, 109.0653; found 109.0659.
*3,5-dimethylphenol* (**2c**)**:** Yield: 97%, 119.6 mg, yellow solid, mp 59–61 °C, R_f_ = 0.62 (H/E = 5:1). ^1^H NMR (500 MHz, CDCl_3_) *δ* 6.64 (s, 1H), 6.52 (s, 2H), 5.12 (brs, 1H), and 2.31 (s, 6H). ^13^C NMR (126 MHz, CDCl_3_) *δ* 155.3, 139.6 (2C), 122.6, 113.1 (2C), and 21.3 (2C). [M + H]^+^ calcd for C8H11O, 123.0810; found 123.0808.
*[1,1′-biphenyl]-4-ol* (**2d**)**:** Yield: 98%, 166.6 mg, light yellow solid, mp 162–164 °C, R_f_ = 0.44 (H/E = 5:1).^1^H NMR (500 MHz, CDCl_3_) *δ* 7.66–7.33 (m, 7H), 6.94 (d, *J* = 6.7 Hz, 2H), and 4.87 (s, 1H). ^13^C NMR (126 MHz, CDCl_3_) *δ* 155.1, 140.8, 134.0, 131.0, 128.8 (2C), 128.4 (2C), 126.7 (2C), and 115.7(2C). [M + H]^+^ calcd for C_12_H_11_O, 171.0810; found 171.0815.
*[1,1′-biphenyl]-2-ol* (**2e**)**:** Yield: 99%, 168.8 mg, light yellow solid, mp 29–31 °C, R_f_ = 0.43 (H/E = 5:1).^1^H NMR (500 MHz, CDCl_3_) *δ* 7.56 (d, *J* = 4.4 Hz, 4H), 7.48–7.45 (m, 1H), 7.34 (d, *J* = 7.4 Hz, 2H), 7.08 (dd, *J* = 12.2, 7.4 Hz, 2H), and 5.40 (s, 1H). ^13^C NMR (126 MHz, CDCl_3_) *δ* 152.5, 137.2, 130.4, 129.32 (2C), 129.25, 129.2, 128.3, 127.9 (2C), 121.0, and 116.0. [M + H]^+^ calcd for C_12_H_11_O, 171.0810; found 171.0818.
4-(methylthio)phenol (**2f**)**:** Yield: 61%, 85.6 mg, white solid, mp 82–84 °C, R_f_ = 0.36 (H/E = 5:1). ^1^H NMR (500 MHz, CDCl_3_) *δ* 7.24 (d, *J* = 8.6 Hz, 2H), 6.81 (d, *J* = 8.6 Hz, 2H), 5.34 (brs, 1H), and 2.46 (s, 3H). ^13^C NMR (126 MHz, CDCl_3_) *δ* 154.1, 130.4 (2C), 128.8, 116.1 (2C), and 18.1. [M + H]^+^ calcd for C_7_H_9_OS, 141.0374; found 141.0369.
2-methoxyphenol (**2g**)**:** Yield: 93%, 115.2 mg, colorless oil, R_f_ = 0.62 (H/E = 5:1). ^1^H NMR (500 MHz, CDCl_3_) *δ* 6.97–6.95 (m, 1H), 6.92–6.88 (m, 3H), 5.67 (brs, 1H), and 3.92 (s, 3H). ^13^C NMR (126 MHz, CDCl_3_) *δ* 146.6, 145.7, 121.5, 120.2, 114.6, 110.7, and 55.9. [M + H]^+^ calcd for C_7_H_9_O_2_, 125.0603; found 125.0605.
4-(heptyloxy)phenol (**2h**)**:** Yield: 94%, 195.6 mg, white solid, mp 56–58 °C, R_f_ = 0.58 (H/E = 5:1). ^1^H NMR (500 MHz, CDCl_3_) δ 6.80 (q, *J* = 9.2 Hz, 4H), 5.34 (brs, 1H), 3.93 (t, *J* = 6.7 Hz, 2H), 1.79 (p, *J* = 6.9 Hz, 2H), 1.53–1.42 (m, 2H), 1.43–1.28 (m, 6H), and 0.93 (t, *J* = 6.7 Hz, 3H). ^13^C NMR (126 MHz, CDCl_3_) δ 153.2, 149.4, 116.1 (2C), 115.8 (2C), 69.0, 31.8, 29.4, 29.1, 26.0, 22.6, and 14.1. [M + H]^+^ calcd for C_13_H_21_O_2_, 209.1542; found 209.1540.
4-phenoxyphenol (**2i**)**:** Yield: 94%, 175.0 mg, light brown solid, mp 74–76 °C, R_f_ = 0.48 (H/E = 5:1). ^1^H NMR (500 MHz, CDCl_3_) δ 7.34 (t, *J* = 7.9 Hz, 2H), 7.09 (t, *J* = 7.3 Hz, 1H), 7.00–6.97 (m, 4H), 6.86 (d, *J* = 8.8 Hz, 2H), and 5.28 (brs, 1H). ^13^C NMR (126 MHz, CDCl_3_) δ 158.4, 151.7, 150.3, 129.7 (2C), 122.7, 121.1 (2C), 117.7 (2C), and 116.5 (2C). [M + H]^+^ calcd for C_12_H_11_O_2_, 187.0759; found 187.0759.
4-(diphenylamino)phenol (**2j**)**:** Yield: 78%, 204.2 mg, white solid, mp 118–120 °C, R_f_ = 0.48 (H/E = 5:1). ^1^H NMR (500 MHz, CDCl_3_) δ 7.26 (t, *J* = 8.6 Hz, 4H), 7.13–6.97 (m, 8H), 6.81 (d, *J* = 8.1 Hz, 2H), and 4.98 (brs, 1H). ^13^C NMR (126 MHz, CDCl_3_) δ 152.1, 148.2(2C), 141.0, 129.1 (4C), 127.6 (2C), 123.0 (4C), 122.0 (2C), and 116.3 (2C). [M + H]^+^ calcd for C_18_H_16_NO, 262.1232; found 262.1237.
*benzo[d]*[1,3]*dioxol-5-ol* (**2k**)**:** Yield: 91%, 125.6 mg, white solid, mp 58–60 °C, R_f_ = 0.38 (H/E = 5:1). ^1^H NMR (500 MHz, CDCl_3_) δ 6.68 (d, *J* = 8.3 Hz, 1H), 6.45 (d, *J* = 2.4 Hz, 1H), 6.28 (dd, *J* = 8.3, 2.4 Hz, 1H), 5.93 (s, 2H), and 4.87 (brs, 1H). ^13^C NMR (126 MHz, CDCl_3_) δ 150.5, 148.3, 141.6, 108.2, 106.8, 101.2, and 98.4. [M + H]^+^ calcd for C_7_H_7_O_3_, 139.0395; found 139.0393.
3-chlorophenol (**2l**)**:** Yield: 98%, 125.8 mg, colorless oil, R_f_ = 0.51 (H/E = 5:1). ^1^H NMR (500 MHz, CDCl_3_) δ 7.18 (t, *J* = 8.1 Hz, 1H), 6.94 (d, *J* = 7.9 Hz, 1H), 6.88 (s, 1H), 6.76–6.74 (m, 1H), and 5.11 (brs, 1H). ^13^C NMR (126 MHz, CDCl_3_) δ 156.3, 134.9, 130.5, 121.1, 115.9, and 113.8. [M + H]^+^ calcd for C_6_H_6_ClO, 129.0107; found 129.0111.
1-(3-hydroxyphenyl)ethan-1-one (**2m**)**:** Yield: 94%, 127.8 mg, yellow solid, mp 92–94 °C, R_f_ = 0.54 = (H/E 5:1). ^1^H NMR (500 MHz, CDCl_3_) δ 7.58 (s, 1H), 7.53 (d, *J* = 7.7 Hz, 1H), 7.36 (t, *J* = 7.9 Hz, 1H), 7.15 (dd, *J* = 8.1, 2.5 Hz, 1H), 6.98 (brs, 1H), and 2.63 (s, 3H). ^13^C NMR (126 MHz, CDCl_3_) δ 199.7, 156.5, 138.3, 130.0, 121.1, 121.0, 114.8, and 26.8. [M + H]^+^ calcd for C_8_H_9_O_2_, 137.0603; found 137.0599.
4-hydroxybenzonitrile (**2n**)**:** Yield: 91%, 108.3 mg, gray solid, mp 110–112 °C, R_f_ = 0.20 (H/E = 5:1). ^1^H NMR (500 MHz, CDCl_3_) δ 7.58 (d, *J* = 8.7 Hz, 2H), 7.22 (brs, 1H), and 6.98 (d, *J* = 8.8 Hz, 2H). ^13^C NMR (126 MHz, CDCl_3_) δ 160.5, 134.4 (2C), 119.3, 116.6 (2C), and 102.8. [M + H]^+^ calcd for C_7_H_6_NO, 120.0449; found 120.0457.
4-nitrophenol (**2o**)**:** Yield: 80%, 111.3 mg, yellow solid, mp 108–110 °C, R_f_ = 0.18 (H/E = 5:1). ^1^H NMR (500 MHz, CDCl_3_) δ 8.20 (d, *J* = 8.8 Hz, 2H), 6.96 (d, *J* = 8.8 Hz, 2H), and 6.14 (s, 1H). ^13^C NMR (126 MHz, CDCl_3_) δ 161.5, 141.6, 126.3 (2C), and 115.8 (2C). [M + H]^+^ calcd for C_6_H_6_NO_3_, 140.0348; found 140.0349.
naphthalen-2-ol (**2p**)**:** Yield: 98%, 141.2 mg, white solid, mp 118–120 °C, R_f_ = 0.48 (H/E = 5:1). ^1^H NMR (500 MHz, CDCl_3_) δ 7.83–7.79 (m, 2H), 7.71 (d, *J* = 7.9 Hz, 1H), 7.48 (t, *J* = 7.1 Hz, 1H), 7.38 (t, *J* = 7.0 Hz, 1H), 7.18–7.14 (m, 2H), and 5.20 (brs, 1H). ^13^C NMR (126 MHz, CDCl_3_) δ 153.3, 134.6, 123.0, 129.0, 127.8, 126.6, 126.5, 123.7, 117.8, and 109.6. [M + H]^+^ calcd for C_10_H_9_O, 145.0653; found 145.0658.
naphthalen-1-ol (**2q**)**:** Yield: 95%, 136.8 mg, white solid, mp 92–94 °C, R_f_ = 0.52 (H/E = 5:1). ^1^H NMR (500 MHz, CDCl_3_) δ 8.24–8.22 (m, 1H), 7.88–7.86 (m, 1H), 7.56–7.49 (m, 3H), 7.35 (t, *J* = 7.8 Hz, 1H), 6.85 (d, *J* = 7.4 Hz, 1H), and 5.35 (brs, 1H). ^13^C NMR (126 MHz, CDCl_3_) δ 151.4, 134.8, 127.7, 126.5, 125.9, 125.3, 124.4, 121.6, 120.8, and 108.7. [M + H]^+^ calcd for C_10_H_9_O, 145.0653; found 145.0655.
6-methoxynaphthalen-2-ol (**2r**)**:** Yield: 90%, 156.8 mg, white solid, mp 146–148 °C, R_f_ = 0.56 (H/E = 5:1). ^1^H NMR (500 MHz, CDCl_3_) δ 7.67 (d, *J* = 8.7 Hz, 1H), 7.61 (d, *J* = 8.8 Hz, 1H), 7.16–7.09 (m, 4H), 4.90 (s, 1H), and 3.93 (s, 3H). ^13^C NMR (126 MHz, CDCl_3_) δ 156.1, 151.8, 129.8, 129.7, 128.5, 127.8, 119.3, 118.1, 109.8, 106.0, and 55.3. [M + H]^+^ calcd for C_11_H_11_O_2_, 175.0759; found 175.0766.
4-(9H-carbazol-9-yl)phenol (**2s**)**:** Yield: 78%, 202.3 mg, white solid, mp 94–96 °C, R_f_ = 0.40 (H/E = 5:1). ^1^H NMR (500 MHz, CDCl_3_) δ 8.25 (t, *J* = 7.2 Hz, 2H), 7.52–7.36 (m, 8H), 7.06 (d, *J* = 8.4 Hz, 2H), and 5.26 (brs, 1H). ^13^C NMR (126 MHz, CDCl_3_) δ 154.8, 141.4 (2C), 130.6, 128.9 (2C), 126.0 (2C), 123.2 (2C), 120.4 (2C), 119.9 (2C), 116.7 (2C), and 109.8 (2C). [M + H]^+^ calcd for C_18_H_14_NO, 260.1075; found 260.1071.
*4-((1s,4r)-4-propylcyclohexyl)phenol* (**2t**)**:** Yield: 98%, 214.1 mg, white solid, mp 44–46 °C, R_f_ = 0.42 (H/E = 5:1). ^1^H NMR (500 MHz, CDCl_3_) δ 7.10 (d, *J* = 8.5 Hz, 2H), 6.78 (d, *J* = 8.5 Hz, 2H), 4.69 (brs, 1H), 2.45–2.40 (m, 1H), 1.89–1.86 (m, 4H), 1.47–1.21 (m, 7H), 1.10–1.02 (m, 2H), and 0.93 (t, *J* = 7.3 Hz, 3H). ^13^C NMR (126 MHz, CDCl_3_) δ 153.5, 140.4, 127.9 (2C), 115.0 (2C), 43.8, 39.8, 37.0, 34.6 (2C), 33.6 (2C), 20.1, and 14.4. [M + H]^+^ calcd for C_15_H_23_O, 219.1749; found 219.1754.

#### 3.2.2. The Typical Procedure for the Oxidative Hydroxylation of Alkylboronic Acids

A mixture of alkylboronic acids **3** (1.0 mmol) and citric acid (0.2 mg, 0.001 mmol, 0.1 mol%) in H_2_O (3 mL) was added into a Schlenk flask (25 mL). Then, 30% aqueous H_2_O_2_ (227 µL, 2.0 equiv.) was added and stirred at room temperature under an air atmosphere for 2 h. After the reaction was finished, the reaction mixture was diluted with water and then extracted with ethyl acetate. The combined organic layer was dried on sodium sulfate and filtered. Then, the solvent evaporated under reduced pressure and the residue was purified by column chromatography (petroleum ether/ethyl acetate 5:1 to 4:1) to provide the product **4**.

*2-phenylethan-1-ol* (**4a**)**:** Yield: 90%, 110.0 mg, colorless oil, R_f_ = 0.40 (H/E = 5:1). ^1^H NMR (500 MHz, CDCl_3_) δ 7.35 (t, *J* = 7.7 Hz, 2H), 7.28–7.25 (m, 3H), 3.89 (t, *J* = 6.6 Hz, 2H), 2.90 (t, *J* = 6.5 Hz, 2H), and 1.54 (brs, 1H). ^13^C NMR (126 MHz, CDCl_3_) δ 138.5, 129.1 (2C), 128.6 (2C), 126.5, 63.7, and 39.2. [M + H]^+^ calcd for C_8_H_11_O, 123.0810; found 123.0807.
*cyclohexanol* (**4b**)**:** Yield: 88%, 88.2 mg, colorless oil, R_f_ = 0.34 (H/E = 4:1). ^1^H NMR (600 MHz, CDCl_3_) δ 3.53–3.50 (m, 1H), 2.94 (s, 1H), 1.82 (dd, *J* = 9.6, 4.8 Hz, 2H), 1.67 (dd, *J* = 9.1, 4.7 Hz, 2H), 1.50–1.46 (m, 1H), 1.22–1.16 (m, 4H), and 1.12–1.07 (m, 1H). ^13^C NMR (151 MHz, CDCl_3_) δ 70.1, 35.4 (2C), 25.4, and 24.2 (2C). [M + H]^+^ calcd for C_6_H_13_O, 101.0966; found 101.0967.
hexan-1-ol (**4c**)**:** Yield: 84%, 85.8 mg, colorless oil, R_f_ = 0.38 (H/E = 4:1). ^1^H NMR (600 MHz, CDCl_3_) δ 4.41 (brs, 1H), 3.30 (t, *J* = 6.9 Hz, 2H), 1.29 (p, *J* = 6.9 Hz, 2H), 1.22–0.95 (m, 6H), and 0.65 (t, *J* = 7.0 Hz, 3H). ^13^C NMR (151 MHz, CDCl_3_) δ 61.8, 32.3, 31.5, 25.3, 22.4, and 13.6. [M + H]^+^ calcd for C_6_H_15_O, 103.1123; found 103.1128.
heptan-1-ol (**4d**)**:** Yield: 84%, 97.6 mg, colorless oil, R_f_ = 0.40 (H/E = 4:1). ^1^H NMR (600 MHz, CDCl_3_) δ 3.64 (t, *J* = 6.7 Hz, 2H), 1.68 (s, 1H), 1.62–1.53 (m, 2H), 1.38–1.26 (m, 8H), and 0.90 (t, *J* = 7.0 Hz, 3H).^13^C NMR (151 MHz, CDCl_3_) δ 63.0, 32.8, 31.8, 29.1, 25.7, 22.6, and 14.1. [M + H]^+^ calcd for C_7_H_17_O, 117.1279; found 117.1277.
decan-1-ol (**4e**)**:** Yield: 90%, 142.4 mg, colorless oil, R_f_ = 0.48 (H/E = 4:1). ^1^H NMR (600 MHz, CDCl_3_) δ 3.64 (t, *J* = 6.7 Hz, 2H), 1.68 (s, 1H), 1.61–1.53 (m, 2H), 1.38–1.25 (m, 14H), and 0.89 (t, *J* = 7.0 Hz, 3H). ^13^C NMR (151 MHz, CDCl_3_) δ 63.0, 32.8, 31.9, 29.64, 29.57, 29.5, 29.3, 25.8, 22.7, and 14.1. [M + H]^+^ calcd for C_10_H_23_O, 159.1749; found 159.1752.

#### 3.2.3. Substrate Extension Studies

Potassium phenyltrifluoroborate (**5**) (184.0 mg, 1.0 mmol, 1.0 equiv.) or a phenylboronic pinacol ester (**6**), 30% aqueous H_2_O_2_ (227 µL, 2.0 equiv.), citric acid (0.2 mg, 0.001 mmol, 0.1 mol%), and water (3 mL) under an air atmosphere were added to an oven-dried 25 mL Schlenk tube equipped with a magnetic stir bar. The reaction mixture was stirred at room temperature for 2 h. After the reaction was finished, the reaction mixture was diluted with water and then extracted with ethyl acetate. The combined organic layer was dried on sodium sulfate and filtered. Then, the solvent was evaporated under reduced pressure and the residue was purified by column chromatography (petroleum ether/ethyl acetate 5:1) to afford the desired product **2a** as a light pink solid.

### 3.3. Large-Scale Synthesis

Phenylboronic acid (**1a**) (2.44 g, 20.0 mmol, 1.0 equiv.), 30% aqueous H_2_O_2_ (4.5 mL, 2.0 equiv.), citric acid (3.8 mg, 0.02 mmol, 0.1 mol%), and water (60 mL) were added to an oven-dried 250 mL round-bottom flask equipped with a magnetic stir bar under an air atmosphere. The reaction mixture was stirred at room temperature for 2 h. After the reaction was finished, the reaction mixture was diluted with water and then extracted with ethyl acetate. The combined organic layer was dried on sodium sulfate and filtered. Then, the solvent was evaporated under reduced pressure, and the residue was purified by column chromatography (petroleum ether/ethyl acetate 5:1) to afford the desired product **2a**.

## 4. Conclusions

In summary, employing a citric acid-catalyzed transition metal-free strategy, we have successfully developed a facile, convenient, and alternative method to achieve the oxidative hydroxylation of organoboron compounds. Advantageously, alcohols can survive under our organocatalytic oxidation conditions. The oxidative hydroxylation reaction of various aromatic and aliphatic boric acids proceeded smoothly by employing H_2_O_2_ as a green oxidant in an aqueous environment at room temperature to produce phenols and aliphatic alcohols in satisfactory to excellent yields (up to 99% yield). Notably, the catalyst citric acid is nontoxic, low-cost, biodegradable, and environmentally benign, and the loading was only 0.1 mol%. The readily available starting materials, the mild and green reaction conditions, and the experimental simplicity make the present methodology highly useful in the synthesis of phenols and aliphatic alcohols. Based on the significance of both environmental protection and resource conservation, we believe that this methodology using natural substances as catalysts and H_2_O as a solvent at room temperature could be of interest to organic chemists.

## Figures and Tables

**Table 1:** Optimization of reaction conditions ^a^.

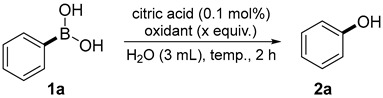				
Entry	Cat. (x mol)	Oxidant (x equiv.)	Temp. (°C)	Yield (%) ^b^
1	citric acid (0.1)	H_2_O_2_ (2.0)	40	93
2	citric acid (0.1)	H_2_O_2_ (2.0)	30	96
3	citric acid (0.1)	H_2_O_2_ (2.0)	rt	98
4	citric acid (0.1)	air	rt	n. d. ^c^
5	citric acid (0.1)	TBHP (2.0)	rt	30
6	citric acid (0.1)	DTBP (2.0)	rt	40
7	citric acid (0.1)	O_2_ (balloon)	rt	n. d.
8	citric acid (0.1)	NaClO_2_ (2.0)	rt	64
9	citric acid (0.1)	H_2_O_2_ (1.0)	rt	68
10	citric acid (0.1)	H_2_O_2_ (3.0)	rt	95
11	citric acid (0.05)	H_2_O_2_ (2.0)	rt	90
12	citric acid (0.2)	H_2_O_2_ (2.0)	rt	97
13 ^d^	citric acid (0.1)	H_2_O_2_ (2.0)	rt	91
14	-	H_2_O_2_ (2.0)	rt	66
15	acetic acid (0.1)	H_2_O_2_ (2.0)	rt	69
16	benzoic acid (0.1)	H_2_O_2_ (2.0)	rt	74
17	tartaric acid (0.1)	H_2_O_2_ (2.0)	rt	83
18	formic acid (0.1)	H_2_O_2_ (2.0)	rt	81

^a^ Reaction conditions: **1a** (94.1 mg, 1.0 mmol), oxidant (x equiv.), and citric acid (0.2 mg, 0.1 mol%) in H_2_O (3 mL) at heating or room temperature for 2 h. ^b^ Isolated yield. ^c^ The desired product was not detected; the starting material was recovered. ^d^ The reaction was performed for 1 h.

## Data Availability

Data obtained in this project are contained within this article and are available upon request from the authors.

## Supplementary Materials

The following supporting information can be downloaded at https://www.mdpi.com/article/10.3390/molecules28237915/s1, Crystallographic data and copies of NMR spectra.
