# Supplementary material for: A Facile and General Oxidative Hydroxylation of Organoboron Compounds: Citric Acid as an Efficient Catalyst in Water to Access Phenolic and Alcoholic Motifs
Source: Molecules. 2023 Dec 3;28(23):7915. doi: 10.3390/molecules28237915 (PMC10708500; doi:10.3390/molecules28237915)

Supporting Information

A Facile and General Oxidative Hydroxylation of Organoboron Compounds: Citric Acid as An Efficient Organocatalyst in Water to Access Phenolic and Alcoholic Motifs

Jia-Hui Zhou,[†,‡] Xia Chen,[‡] Dan Yang,[‡] Chun-Yan Liu,[†] Xiao-Yu Zhou*,[‡]

[†]College of Environmental and Chemical Engineering, Dalian University, Dalian 116622, China

[‡]School of Chemistry and Materials Engineering, Liupanshui Normal University, Liupanshui 553004, China

Table of Contents

1. General and Materials……………………………………………………..………………S3

2. Screen of Catalysts for the Synthesis of Phenols……………………………………..…S3

3. The Typical Procedure for the Oxidative Hydroxylation of Arylboronic Acids……..…S4-8

4. The Typical Procedure for the Oxidative Hydroxylation of Alkylboronic Acids………S8-9

5. Substrate Extension Studies…………………………………………………….……S9-10

6. Large–scale Synthesis………………………………………………………….......S10

7. Copy of NMR for the Products……………………………….……..…...……….….S11-35

1. General Information.

Unless otherwise noted, all reactions were carried out in oven-dried 25-mL Schlenk tubes under a nitrogen atmosphere. IKA plate was used as the heat source. All reagents and solvents were of pure analytical grade. Thin layer chromatography (TLC) was performed on HSGF254 silica gel, pre-coated on glass-backed plates coated with 0.2 mm silica and revealed with either a UV lamp (λ_{max} = 254 nm). The products were purified by flash column chromatography on silica gel 200-300 mesh. ^{1}H and ^{13}C NMR spectra were recorded on a Varian Inova-500 spectrometer (500 MHz for ^{1}H, 126 MHz for ^{13}C), a Bruker Avance NEO 600M NMR Spectrometer (600 MHz for ^{1}H, 151 MHz for ^{13}C) using $CDCl_3$ as the solvent with tetramethylsilane (TMS) as the internal standard at room temperature. The chemical shifts are reported in ppm downfield (δ) from TMS, the coupling constants J are given in Hz. The peak patterns are indicated as follows: s, singlet; d, doublet; dd, doublet of doublet; t, triplet; q, quartet; p, quintet; h, sextet; m, multiplet. High resolution mass spectra were recorded on either a Q-TOF mass spectrometry or a LTQ Orbitrap XL mass spectrometry.

2. Screen of Catalysts for the Synthesis of phenols.

Table S1. Optimization study of catalysts[a]

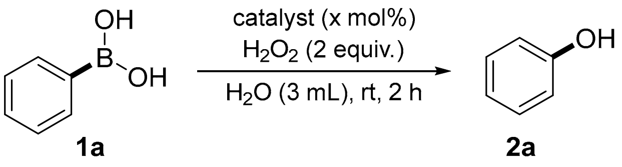

Entry	Catalyst (x mol%)	Yield (%)[b]
1	citric acid (0.1)	98
2	acetic acid (0.1)	69
3	benzoic acid (0.1)	74
4	tartaric acid (0.1)	83
5	formic acid (0.1)	81
6	-	66
7	citric acid (0.05)	90
8	citric acid (0.2)	97

[a] Reaction conditions: **1a** (94.1 mg, 1.0 mmol), H_2O_2 (2.0 mmol, 2 equiv.), and catalyst (x mol%) in H_2O (3 mL) at room temperature under an air atmosphere for 2 h. [b]Isolated yield.

3. The Typical Procedure for the Oxidative Hydroxylation of Arylboronic Acids.

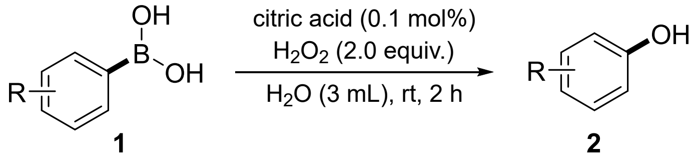

A mixture of arylboronic acids **1** (1.0 mmol), and citric acid (0.2 mg, 0.001 mmol, 0.1 mol%) in H_2O (3 mL) was added into a Schlenk flask (25 mL). Then 30% aqueous H_2O_2 (227 μL, 2.0 eq.) was added and stirred at room temperature under an air atmosphere for 2 hours. After the reaction was finished, the reaction mixture was diluted with water and then extracted with ethyl acetate. The combined organic layer was dried on sodium sulfate and filtered. Then the solvent was evaporated under reduced pressure and the residue was purified by column chromatography (petroleum ether/ethyl acetate 5:1 to 2:1) to provide the product **2**.

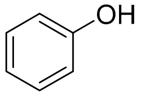

phenol (2a): Yield: 98%, 92.2 mg, light pink solid, mp 38-40 ºC, R_f= 0.50 (H/E = 5:1). ^{1}H NMR (500 MHz, $CDCl_3$) δ 7.29 (d, J = 6.2 Hz, 2H), 6.97 (t, J = 7.4 Hz, 1H), 6.87 (d, J = 7.7 Hz, 2H), 4.64 (brs, 1H). ^{13}C NMR (126 MHz, $CDCl_3$) δ 155.5, 129.7, 120.8, 115.3. $[M + H]^+$ calcd for C6H7O, 95.0497; found 95.0493.

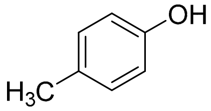

***p*-cresol (2b):** Yield: 98%, 105.7 mg, light brown solid, mp 30-32 ºC, R_f= 0.52 (H/E = 5:1). ^{1}H NMR (500 MHz, $CDCl_3$) δ 7.06 (d, J = 8.0 Hz, 2H), 6.76 (d, J = 8.2 Hz, 2H), 4.72 (brs, 1H), 2.30 (s, 3H). ^{13}C NMR (126 MHz, $CDCl_3$) δ 153.2, 131.0, 130.1, 115.1, 20.5. $[M + H]^+$ calcd for C7H9O, 109.0653; found 109.0659.

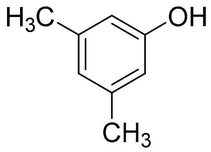

3,5-dimethylphenol (2c): Yield: 97%, 119.6 mg, yellow solid, mp 59-61 ºC, R_f= 0.62 (H/E = 5:1). ^{1}H NMR (500 MHz, $CDCl_3$) δ 6.64 (s, 1H), 6.52 (s, 2H), 5.12 (brs, 1H), 2.31 (s, 6H). ^{13}C NMR (126 MHz, $CDCl_3$) δ 155.3, 139.6, 122.6, 113.1, 21.3. $[M + H]^+$ calcd for C8H11O, 123.0810; found 123.0808.

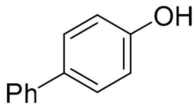

[1,1'-biphenyl]-4-ol (2d): Yield: 98%, 166.6 mg, light yellow solid, mp 162-164 ºC, R_f= 0.44 (H/E = 5:1). ^{1}H NMR (500 MHz, $CDCl_3$) δ 7.58 – 7.34 (m, 7H), 6.94 (d, J = 6.7 Hz, 2H), 4.87 (s, 1H).

^{13}C NMR (126 MHz, $CDCl_3$) δ 155.1, 140.8, 134.0, 131.0, 128.8, 128.4, 126.7, 115.7. $[M + H]^+$ calcd for C12H11O, 171.0810; found 171.0815.

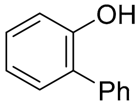

[1,1'-biphenyl]-2-ol (2e): Yield: 99%, 168.8 mg, light yellow solid, mp 29-31 ºC, R_f = 0.43 (H/E = 5:1). ^1H NMR (500 MHz, $CDCl_3$) δ 7.56 (d, J = 4.4 Hz, 4H), 7.48 – 7.45 (m, 1H), 7.34 (d, J = 7.4 Hz, 2H), 7.08 (dd, J = 12.2, 7.4 Hz, 2H), 5.40 (s, 1H). ^{13}C NMR (126 MHz, $CDCl_3$) δ 152.5, 137.2, 130.4, 129.32, 129.25, 129.2, 128.3, 127.9, 121.0, 116.0. $[M + H]^+$ calcd for C12H11O, 171.0810; found 171.0818.

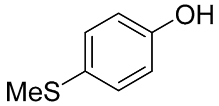

4-(methylthio)phenol (2f): Yield: 61%, 85.6 mg, white solid, mp 82-84 ºC, R_f = 0.36 (H/E = 5:1). ^1H NMR (500 MHz, $CDCl_3$) δ 7.24 (d, J = 8.6 Hz, 2H), 6.81 (d, J = 8.6 Hz, 2H), 5.34 (brs, 1H), 2.46 (s, 3H). ^{13}C NMR (126 MHz, $CDCl_3$) δ 154.1, 130.4, 128.8, 116.1, 18.1. $[M + H]^+$ calcd for C7H9OS, 141.0374; found 141.0369.

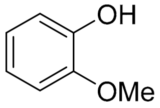

2-methoxyphenol (2g): Yield: 93%, 115.2 mg, colorless oil, R_f = 0.62 (H/E = 5:1). ^1H NMR (500 MHz, $CDCl_3$) δ 6.97-6.95 (m, 1H), 6.92 – 6.88 (m, 3H), 5.67 (brs, 1H), 3.92 (s, 3H). ^{13}C NMR (126 MHz, $CDCl_3$) δ 146.6, 145.7, 121.5, 120.2, 114.6, 110.7, 55.9. $[M + H]^+$ calcd for C7H9O2, 125.0603; found 125.0605.

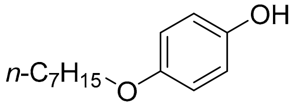

4-(heptyloxy)phenol (2h): Yield: 94%, 195.6 mg, white solid, mp 56-58 ºC, R_f = 0.58 (H/E = 5:1). ^1H NMR (500 MHz, $CDCl_3$) δ 6.80 (q, J = 9.2 Hz, 4H), 5.34 (brs, 1H), 3.93 (t, J = 6.7 Hz, 2H), 1.79 (p, J = 6.9 Hz, 2H), 1.53 – 1.42 (m, 2H), 1.43 – 1.28 (m, 6H), 0.93 (t, J = 6.7 Hz, 3H). ^{13}C NMR (126 MHz, $CDCl_3$) δ 153.2, 149.4, 116.1, 115.8, 69.0, 31.8, 29.4, 29.1, 26.0, 22.6, 14.1. $[M + H]^+$ calcd for C13H21O2, 209.1542; found 209.1540.

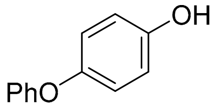

4-phenoxyphenol (2i): Yield: 94%, 175.0 mg, light brown solid, mp 74-76 ºC, R_f = 0.48 (H/E = 5:1). ^1H NMR (500 MHz, $CDCl_3$) δ 7.34 (t, J = 7.9 Hz, 2H), 7.09 (t, J = 7.3 Hz, 1H), 7.00 – 6.97 (m, 4H), 6.86 (d, J = 8.8 Hz, 2H), 5.28 (brs, 1H). ^{13}C NMR (126 MHz, $CDCl_3$) δ 158.4, 151.7, 150.3, 129.7, 122.7, 121.1, 117.7, 116.5. $[M + H]^+$ calcd for C12H11O2, 187.0759; found 187.0759.

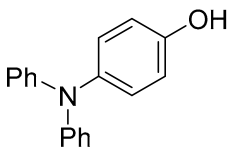

4-(diphenylamino)phenol (2j): Yield: 78%, 204.2 mg, white solid, mp 118-120 °C, R_f = 0.48 (H/E = 5:1). ^{1}H NMR (500 MHz, $CDCl_3$) δ 7.26 (t, *J* = 8.6 Hz, 4H), 7.13 – 6.97 (m, 8H), 6.81 (d, *J* = 8.1 Hz, 2H), 4.98 (brs, 1H). ^{13}C NMR (126 MHz, $CDCl_3$) δ 152.1, 148.2, 141.0, 129.1, 127.6, 123.0, 122.0, 116.3. $[M + H]^+$ calcd for C18H16NO, 262.1232; found 262.1237.

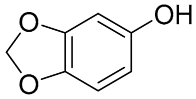

benzo[*d*][1,3]dioxol-5-ol (2k): Yield: 91%, 125.6 mg, white solid, mp 58-60 °C, R_f = 0.38 (H/E = 5:1). ^{1}H NMR (500 MHz, $CDCl_3$) δ 6.68 (d, *J* = 8.3 Hz, 1H), 6.45 (d, *J* = 2.4 Hz, 1H), 6.28 (dd, *J* = 8.3, 2.4 Hz, 1H), 5.93 (s, 2H), 4.87 (brs, 1H). ^{13}C NMR (126 MHz, $CDCl_3$) δ 150.5, 148.3, 141.6, 108.2, 106.8, 101.2, 98.4. $[M + H]^+$ calcd for C7H7O3, 139.0395; found 139.0393.

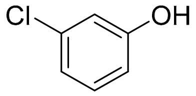

3-chlorophenol (2l): Yield: 98%, 125.8 mg, colorless oil, R_f = 0.51 (H/E = 5:1). ^{1}H NMR (500 MHz, $CDCl_3$) δ 7.18 (t, *J* = 8.1 Hz, 1H), 6.94 (d, *J* = 7.9 Hz, 1H), 6.88 (s, 1H), 6.76 – 6.74 (m, 1H), 5.11 (brs, 1H). ^{13}C NMR (126 MHz, $CDCl_3$) δ 156.3, 134.9, 130.5, 121.1, 115.9, 113.8. $[M + H]^+$ calcd for C6H6ClO, 129.0107; found 129.0111.

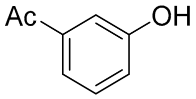

1-(3-hydroxyphenyl)ethan-1-one (2m): Yield: 94%, 127.8 mg, yellow solid, mp 92-94 °C, R_f = 0.54 = (H/E 5:1). ^{1}H NMR (500 MHz, $CDCl_3$) δ 7.58 (s, 1H), 7.53 (d, *J* = 7.7 Hz, 1H), 7.36 (t, *J* = 7.9 Hz, 1H), 7.15 (dd, *J* = 8.1, 2.5 Hz, 1H), 6.98 (brs, 1H), 2.63 (s, 3H). ^{13}C NMR (126 MHz, $CDCl_3$) δ 199.7, 156.5, 138.3, 130.0, 121.1, 121.0, 114.8, 26.8. $[M + H]^+$ calcd for C8H9O2, 137.0603; found 137.0599.

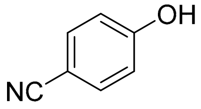

4-hydroxybenzonitrile (2n): Yield: 91%, 108.3 mg, gray solid, mp 110-112 °C, R_f = 0.20 (H/E = 5:1). ^{1}H NMR (500 MHz, $CDCl_3$) δ 7.58 (d, *J* = 8.7 Hz, 2H), 7.22 (brs, 1H), 6.98 (d, *J* = 8.8 Hz, 2H). ^{13}C NMR (126 MHz, $CDCl_3$) δ 160.5, 134.4, 119.3, 116.6, 102.8. $[M + H]^+$ calcd for C7H6NO, 120.0449; found 120.0457.

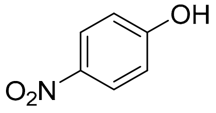

4-nitrophenol (2o): Yield: 80%, 111.3 mg, yellow solid, mp 108-110 °C, R_f = 0.18 (H/E = 5:1). ^{1}H

NMR (500 MHz, $CDCl_3$) δ 8.20 (d, *J* = 8.8 Hz, 2H), 6.96 (d, *J* = 8.8 Hz, 2H), 6.14 (s, 1H). ^{13}C NMR (126 MHz, $CDCl_3$) δ 161.5, 141.6, 126.3, 115.8. $[M + H]^+$ calcd for C6H6NO3, 140.0348; found 140.0349.

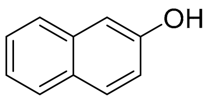

naphthalen-2-ol (2p): Yield: 98%, 141.2 mg, white solid, mp 118-120 ºC, R_f = 0.48 (H/E = 5:1). ^{1}H NMR (500 MHz, $CDCl_3$) δ 7.83 – 7.79 (m, 2H), 7.71 (d, *J* = 7.9 Hz, 1H), 7.48 (t, *J* = 7.1 Hz, 1H), 7.38 (t, *J* = 7.0 Hz, 1H), 7.18 – 7.14 (m, 2H), 5.20 (brs, 1H). ^{13}C NMR (126 MHz, $CDCl_3$) δ 153.3, 134.6, 123.0, 129.0, 127.8, 126.6, 126.5, 123.7, 117.8, 109.6. $[M + H]^+$ calcd for C10H9O, 145.0653; found 145.0658.

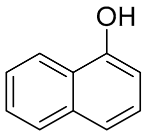

naphthalen-1-ol (2q): Yield: 95%, 136.8 mg, white solid, mp 92-94 ºC, R_f = 0.52 (H/E = 5:1). ^{1}H NMR (500 MHz, $CDCl_3$) δ 8.24 – 8.22 (m, 1H), 7.88 – 7.86 (m, 1H), 7.56 – 7.49 (m, 3H), 7.35 (t, *J* = 7.8 Hz, 1H), 6.85 (d, *J* = 7.4 Hz, 1H), 5.35 (brs, 1H). ^{13}C NMR (126 MHz, $CDCl_3$) δ 151.4, 134.8, 127.7, 126.5, 125.9, 125.3, 124.4, 121.6, 120.8, 108.7. $[M + H]^+$ calcd for C10H9O, 145.0653; found 145.0655.

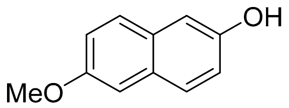

6-methoxynaphthalen-2-ol (2r): Yield: 90%, 156.8 mg, white solid, mp 146-148 ºC, R_f = 0.56 (H/E = 5:1). ^{1}H NMR (500 MHz, $CDCl_3$) δ 7.67 (d, *J* = 8.7 Hz, 1H), 7.61 (d, *J* = 8.8 Hz, 1H), 7.16 – 7.09 (m, 4H), 4.90 (s, 1H), 3.93 (s, 3H). ^{13}C NMR (126 MHz, $CDCl_3$) δ 156.1, 151.8, 129.8, 129.7, 128.5, 127.8, 119.3, 118.1, 109.8, 106.0, 55.3. $[M + H]^+$ calcd for C11H11O2, 175.0759; found 175.0766.

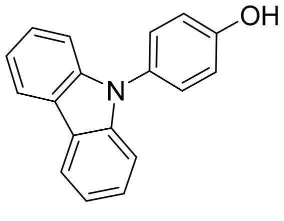

4-(9*H*-carbazol-9-yl)phenol (2s): Yield: 78%, 202.3 mg, white solid, mp 94-96 ºC, R_f = 0.40 (H/E = 5:1). ^{1}H NMR (500 MHz, $CDCl_3$) δ 8.25 (t, *J* = 7.2 Hz, 2H), 7.52 – 7.36 (m, 8H), 7.06 (d, *J* = 8.4 Hz, 2H), 5.26 (brs, 1H). ^{13}C NMR (126 MHz, $CDCl_3$) δ 154.8, 141.4, 130.6, 128.9, 126.0, 123.2, 120.4, 119.9, 116.7, 109.8. $[M + H]^+$ calcd for C18H14NO, 260.1075; found 260.1071.

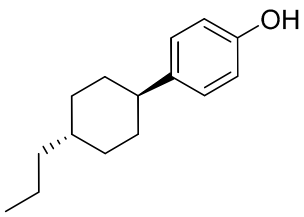

4-((1s,4r)-4-propylcyclohexyl)phenol (2t): Yield: 98%, 214.1 mg, white solid, mp 44-46 ºC, R_f =0.42 (H/E = 5:1). ^{1}H NMR (500 MHz, $CDCl_3$) δ 7.10 (d, *J* = 8.5 Hz, 2H), 6.78 (d, *J* = 8.5 Hz, 2H), 4.69 (brs, 1H), 2.45 – 2.40 (m, 1H), 1.89 – 1.86 (m, 4H), 1.47 – 1.21 (m, 7H), 1.10-1.02 (m, 2H), 0.93 (t, *J* = 7.3 Hz, 3H). ^{13}C NMR (126 MHz, $CDCl_3$) δ 153.5, 140.4, 127.9, 115.0, 43.8, 39.8, 37.0, 34.6, 33.6, 20.1, 14.4. $[M + H]^+$ calcd for C15H23O, 219.1749; found 219.1754.

4. The Typical Procedure for the Oxidative Hydroxylation of Alkylboronic Acids.

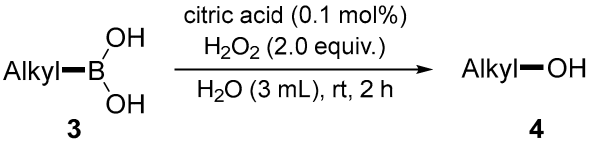

A mixture of alkylboronic acids **3** (1.0 mmol), and citric acid (0.2 mg, 0.001 mmol, 0.1 mol%) in H_2O (3 mL) was added into a Schlenk flask (25 mL). Then 30% aqueous H_2O_2 (227 μL, 2.0 equiv.) was added and stirred at room temperature under an air atmosphere for 2 hours. After the reaction was finished, the reaction mixture was diluted with water and then extracted with ethyl acetate. The combined organic layer was dried on sodium sulfate and filtered. Then the solvent was evaporated under reduced pressure and the residue was purified by column chromatography (petroleum ether/ethyl acetate 5:1 to 4:1) to provide the product **4**.

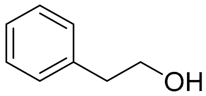

2-phenylethan-1-ol (4a): Yield: 90%, 110.0 mg, colorless oil, R_f= 0.40 (H/E = 5:1). ^{1}H NMR (500 MHz, $CDCl_3$) δ 7.35 (t, *J* = 7.7 Hz, 2H), 7.28 – 7.25 (m, 3H), 3.89 (t, *J* = 6.6 Hz, 2H), 2.90 (t, *J* = 6.5 Hz, 2H),1.54 (brs, 1H). ^{13}C NMR (126 MHz, $CDCl_3$) δ 138.5, 129.1, 128.6, 126.5, 63.7, 39.2. $[M + H]^+$ calcd for C8H11O, 123.0810; found 123.0807.

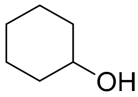

cyclohexanol (4b): Yield: 88%, 88.2 mg, colorless oil, R_f= 0.34 (H/E = 4:1). ^{1}H NMR (600 MHz, $CDCl_3$) δ 3.53-3.50 (m, 1H), 2.94 (s, 1H), 1.82 (dd, *J* = 9.6, 4.8 Hz, 2H), 1.67 (dd, *J* = 9.1, 4.7 Hz, 2H), 1.50-1.46 (m, 1H), 1.22-1.16 (m, 4H), 1.12-1.07 (m, 1H). ^{13}C NMR (151 MHz, $CDCl_3$) δ 70.1, 35.4, 25.4, 24.2. $[M + H]^+$ calcd for C6H13O, 101.0966; found 101.0967.

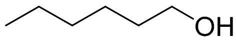

hexan-1-ol (4c): Yield: 84%, 85.8 mg, colorless oil, R_f= 0.38 (H/E = 4:1). ^{1}H NMR (600 MHz, $CDCl_3$) δ 4.41 (brs, 1H), 3.30 (t, *J* = 6.9 Hz, 2H), 1.29 (p, *J* = 6.9 Hz, 2H), 1.22 – 0.95 (m, 6H), 0.65

(t, J = 7.0 Hz, 3H). ^{13}C NMR (151 MHz, $CDCl_3$) δ 61.8, 32.3, 31.5, 25.3, 22.4, 13.6. $[M + H]^+$ calcd for C6H15O, 103.1123; found 103.1128.

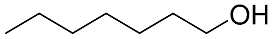

heptan-1-ol (4d): Yield: 84%, 97.6 mg, colorless oil, R_f = 0.40 (H/E = 4:1). ^{1}H NMR (600 MHz, $CDCl_3$) δ 3.64 (t, J = 6.7 Hz, 2H), 1.68 (s, 1H), 1.62 – 1.53 (m, 2H), 1.38 – 1.26 (m, 8H), 0.90 (t, J = 7.0 Hz, 3H). ^{13}C NMR (151 MHz, $CDCl_3$) δ 63.0, 32.8, 31.8, 29.1, 25.7, 22.6, 14.1. $[M + H]^+$ calcd for C7H17O, 117.1279; found 117.1277.

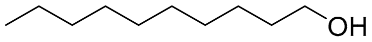

decan-1-ol (4e): Yield: 90%, 142.4 mg, colorless oil, R_f = 0.48 (H/E = 4:1). ^{1}H NMR (600 MHz, $CDCl_3$) δ 3.64 (t, J = 6.7 Hz, 2H), 1.68 (s, 1H), 1.61 – 1.53 (m, 2H), 1.38 – 1.25 (m, 14H), 0.89 (t, J = 7.0 Hz, 3H). ^{13}C NMR (151 MHz, $CDCl_3$) δ 63.0, 32.8, 31.9, 29.64, 29.57, 29.5, 29.3, 25.8, 22.7, 14.1. $[M + H]^+$ calcd for C10H23O, 159.1749; found 159.1752.

5. Substrate Extension Studies.

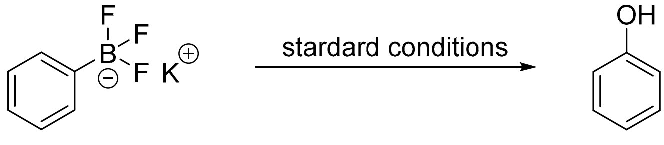

(1)

5 **2a**, 70% yield

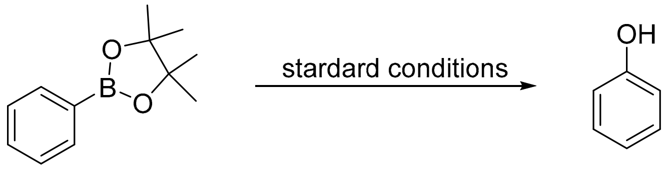

(2)

6 **2a**, 87% yield

Method A: To an oven-dried 25 mL Schlenk tube equipped with a magnetic stir bar was added potassium phenyltrifluoroborate (**5**) (184.0 mg, 1.0 mmol, 1.0 equiv.), 30% aqueous H_2O_2 (227 μL, 2.0 equiv.), citric acid (0.2 mg, 0.001 mmol, 0.1 mol%), and water (3 mL) under an air atmosphere. The reaction mixture was stirred at room temperature for 2 hours. After the reaction was finished, the reaction mixture was diluted with water and then extracted with ethyl acetate. The combined organic layer was dried on sodium sulfate and filtered. Then the solvent was evaporated under reduced pressure and the residue was purified by column chromatography (petroleum ether/ethyl acetate 5:1) to afford the desired product **2a** as a light pink solid (65.9 mg, yield: 70%).

Method B: To an oven-dried 25 mL Schlenk tube equipped with a magnetic stir bar was added phenylboronic pinacol ester (**6**) (204.1 mg, 1.0 mmol, 1.0 equiv.), 30% aqueous H_2O_2 (227 μL, 2.0 equiv.), citric acid (0.2 mg, 0.001 mmol, 0.1 mol%), and water (3 mL) under an air atmosphere. The reaction mixture was stirred at room temperature for 2 hours. After the reaction was finished, the reaction mixture was diluted with water and then extracted with ethyl acetate. The combined organic layer was dried on sodium sulfate and filtered. Then the solvent was evaporated under reduced pressure and the residue was purified by column chromatography (petroleum ether/ethyl acetate 5:1)

to afford the desired product **2a** as a light pink solid (81.8 mg, yield: 87%).

6. Large–scale Synthesis.

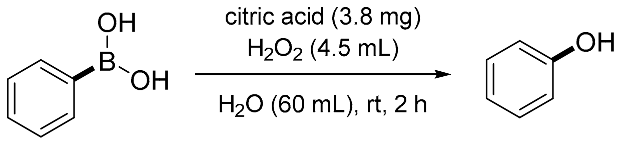

1a, 2.44 g **2a**, 1.73 g

To an oven-dried 250 mL round-bottom flask equipped with a magnetic stir bar was added phenylboronic acid (**1a**) (2.44 g, 20.0 mmol, 1.0 equiv.), 30% aqueous H_2O_2 (4.5 mL, 2.0 equiv.), citric acid (3.8 mg, 0.02 mmol, 0.1 mol%), and water (60 mL) under an air atmosphere. The reaction mixture was stirred at room temperature for 2 hours. After the reaction was finished, the reaction mixture was diluted with water and then extracted with ethyl acetate. The combined organic layer was dried on sodium sulfate and filtered. Then the solvent was evaporated under reduced pressure and the residue was purified by column chromatography (petroleum ether/ethyl acetate 5:1) to afford the desired product **2a**.

7. Copy of NMR for the Products

^{1}H NMR of 2a

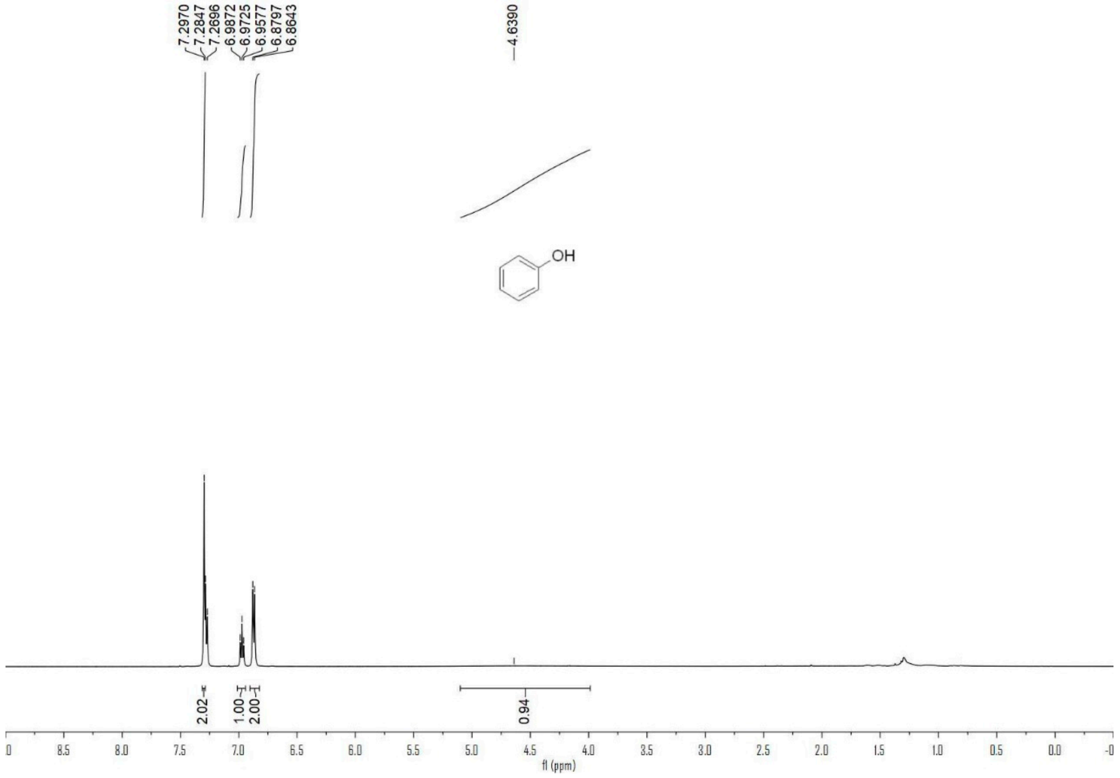

^{13}C NMR of 2a

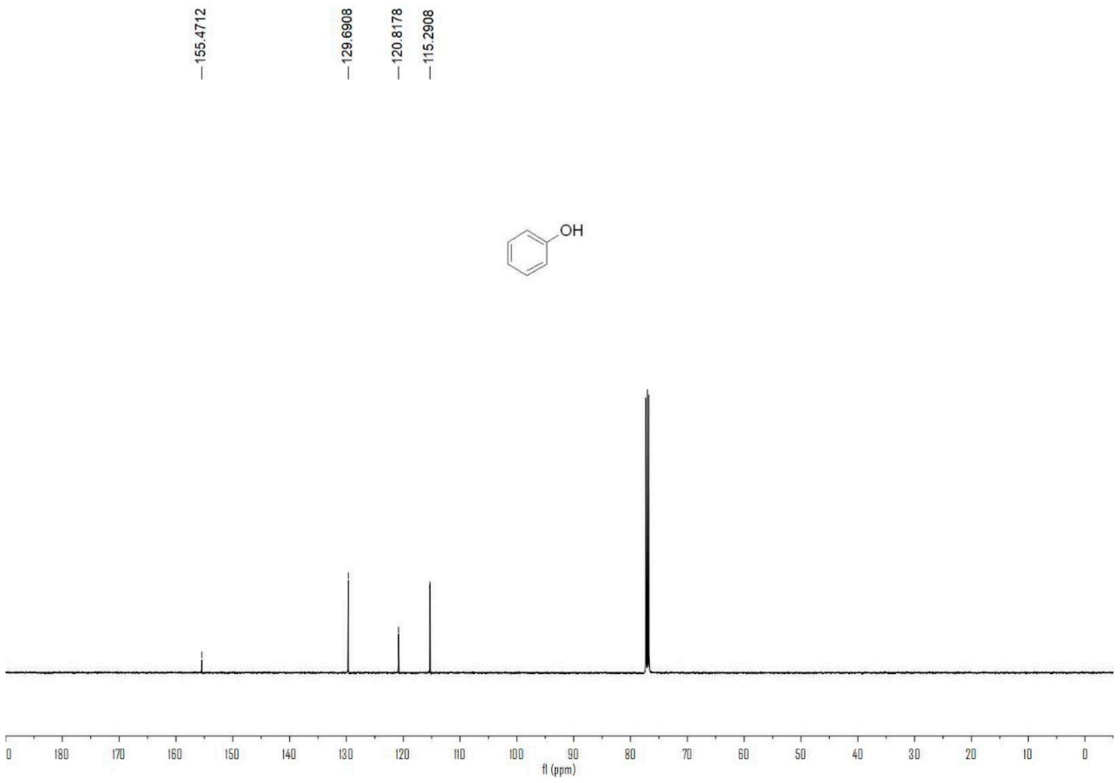

^{1}H NMR of 2b

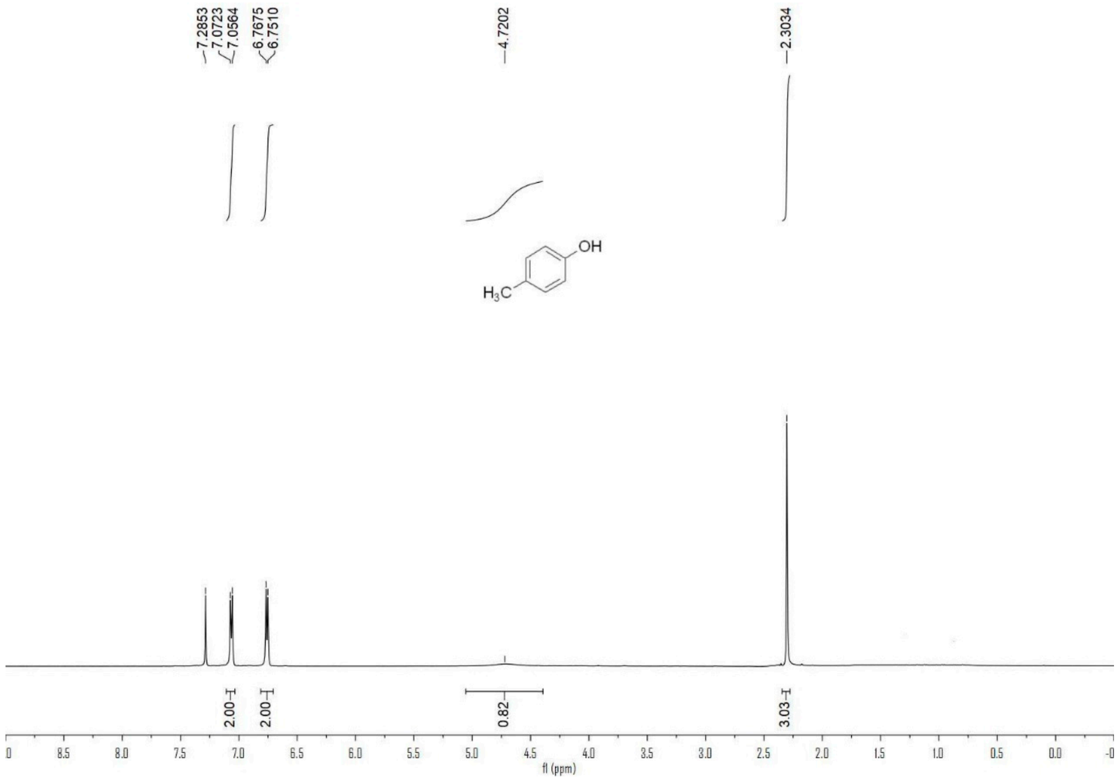

^{13}C NMR of 2b

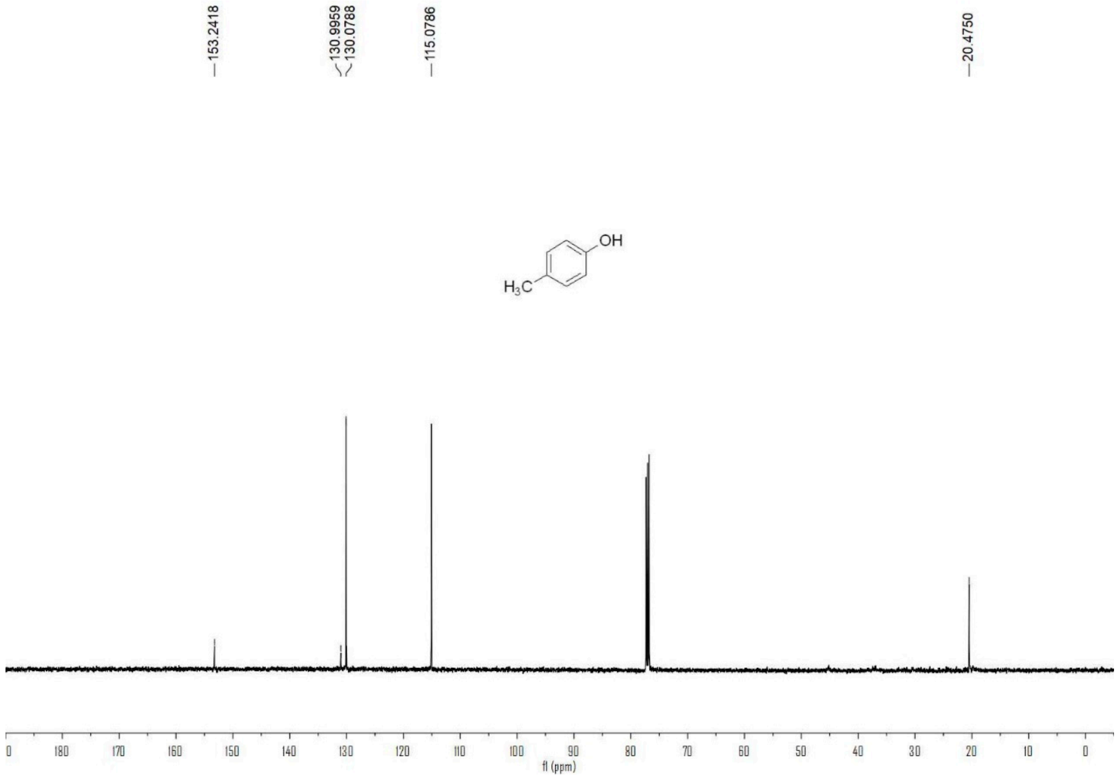

^{1}H NMR of 2c

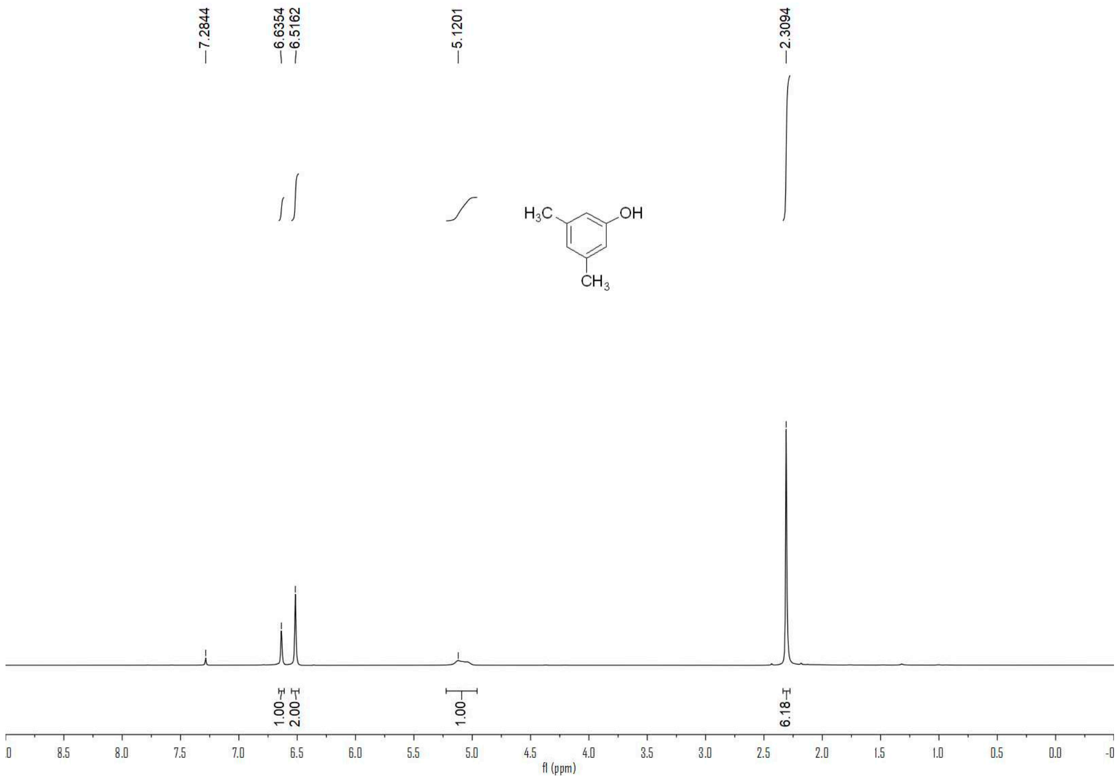

^{13}C NMR of 2c

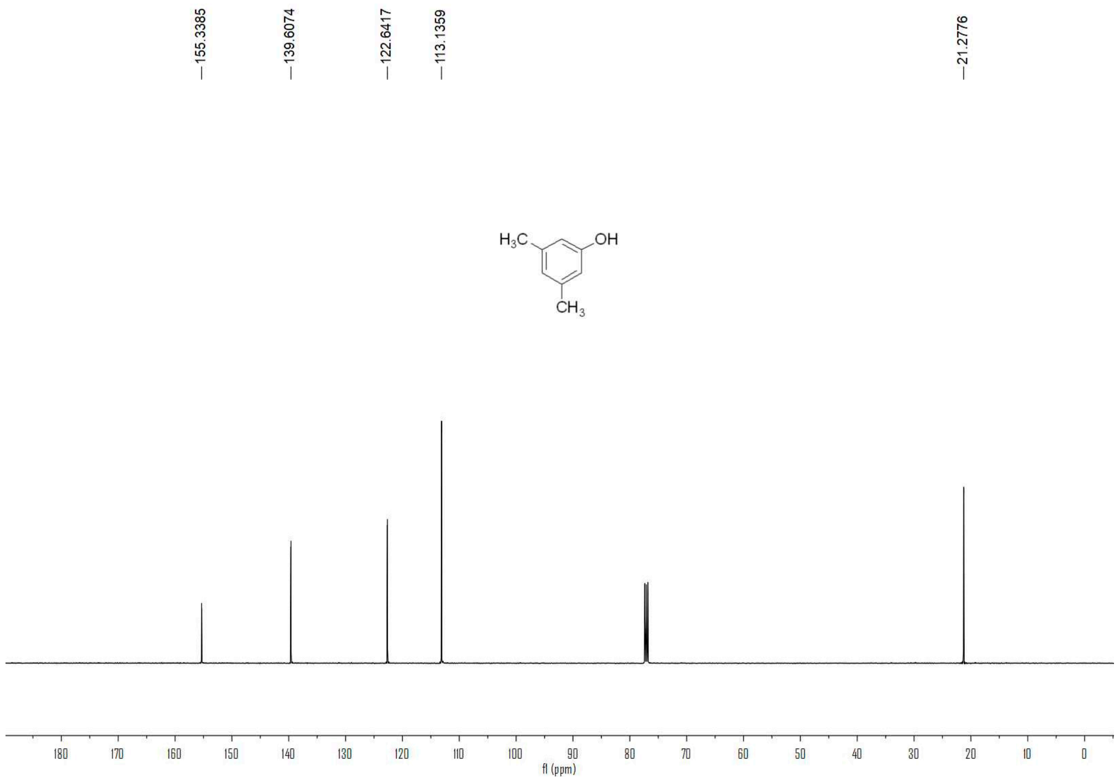

^{1}H NMR of 2d

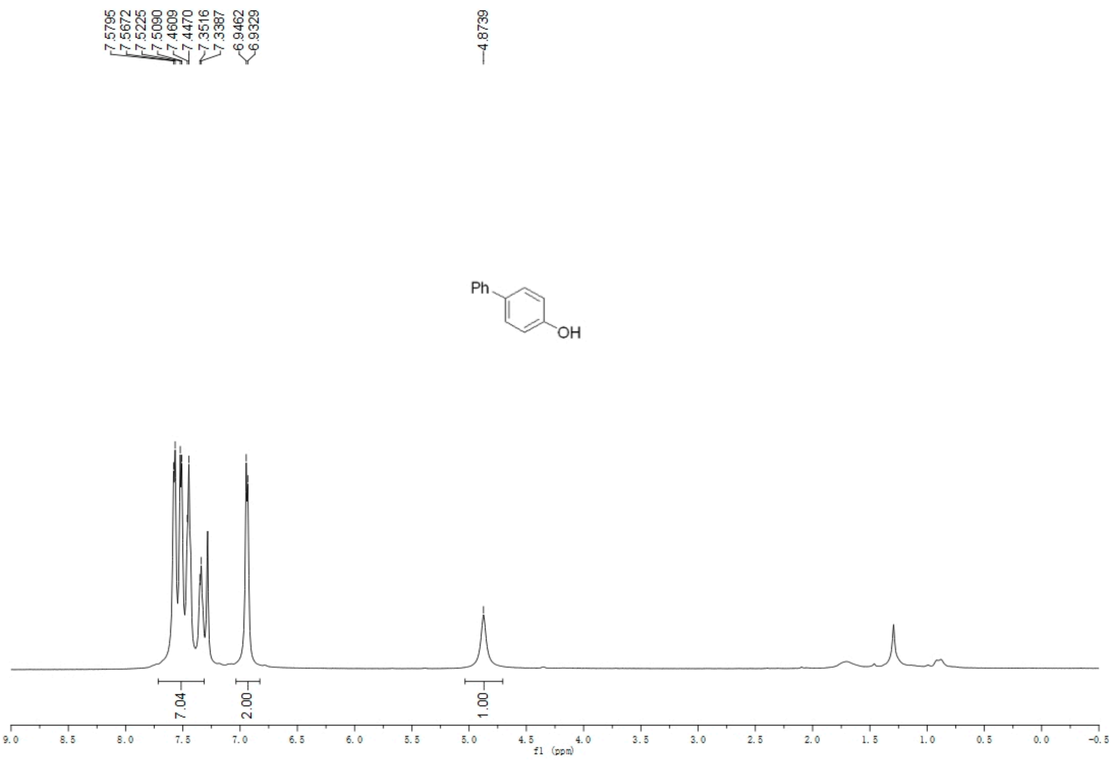

^{13}C NMR of 2d

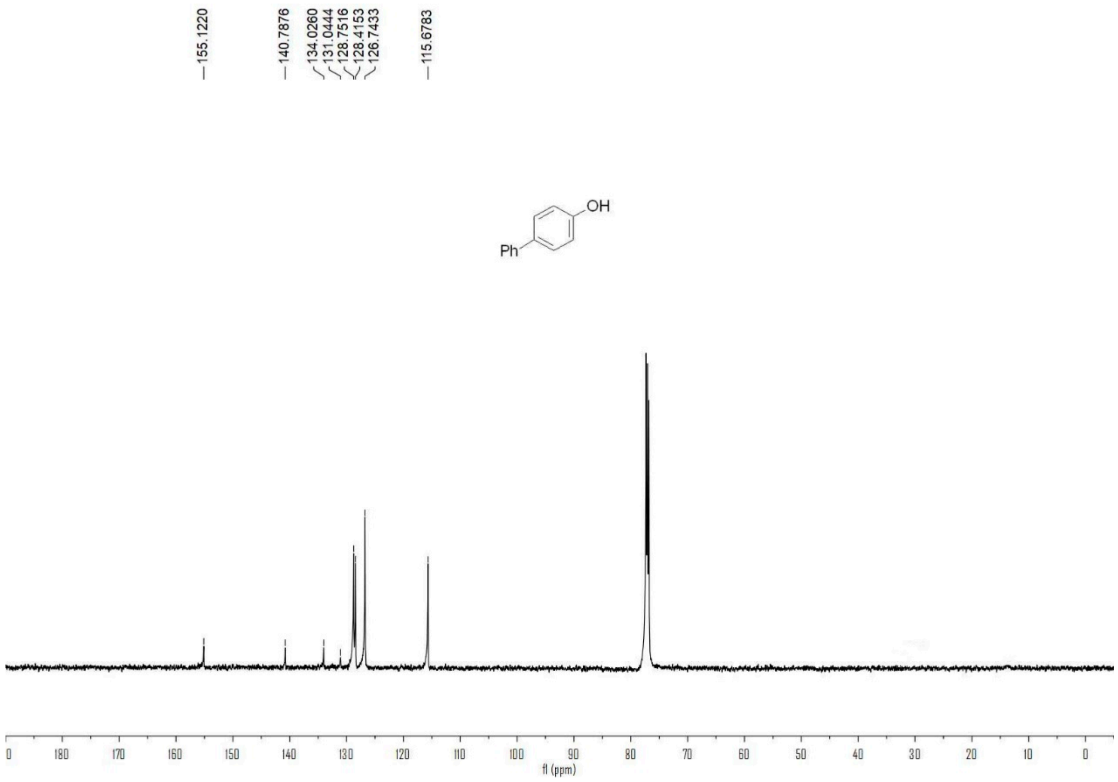

^{1}H NMR of 2e

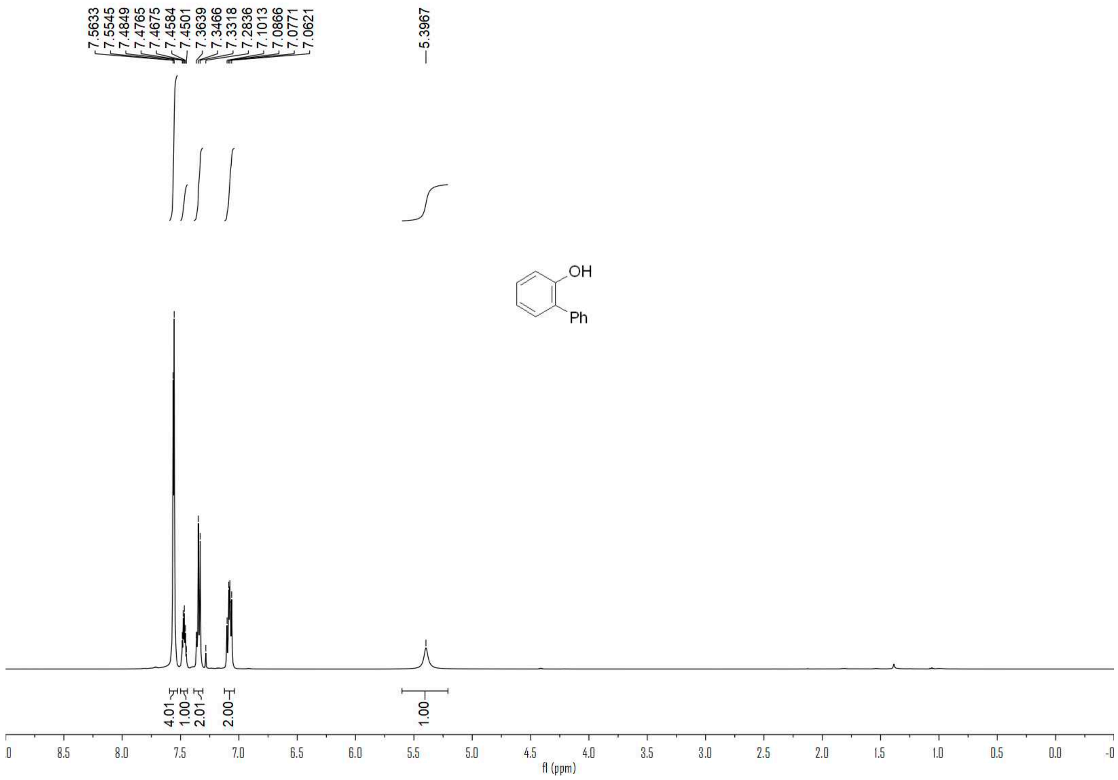

^{13}C NMR of 2e

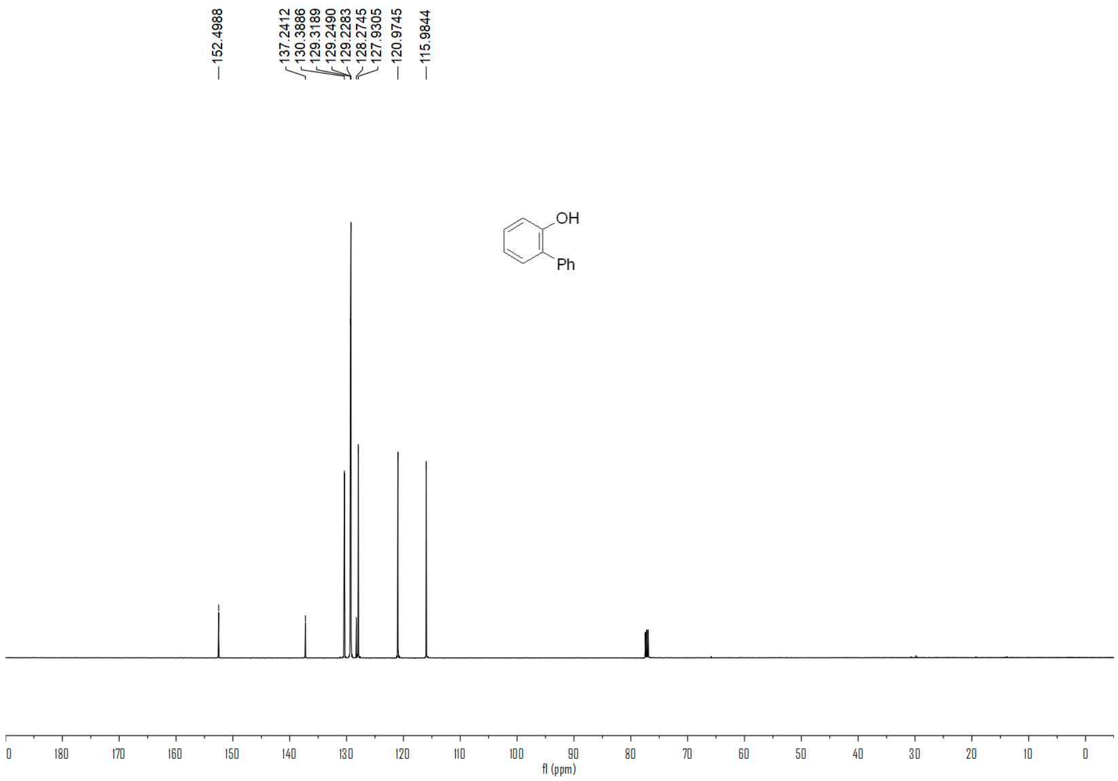

^{1}H NMR of 2f

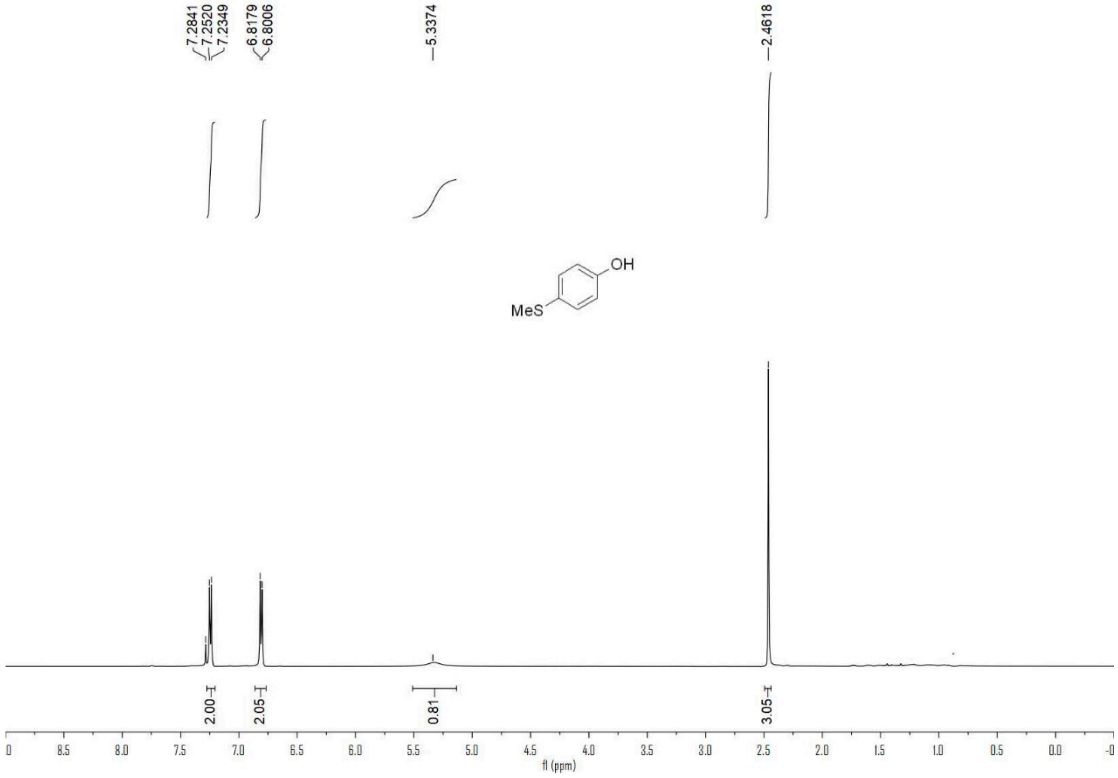

^{13}C NMR of 2f

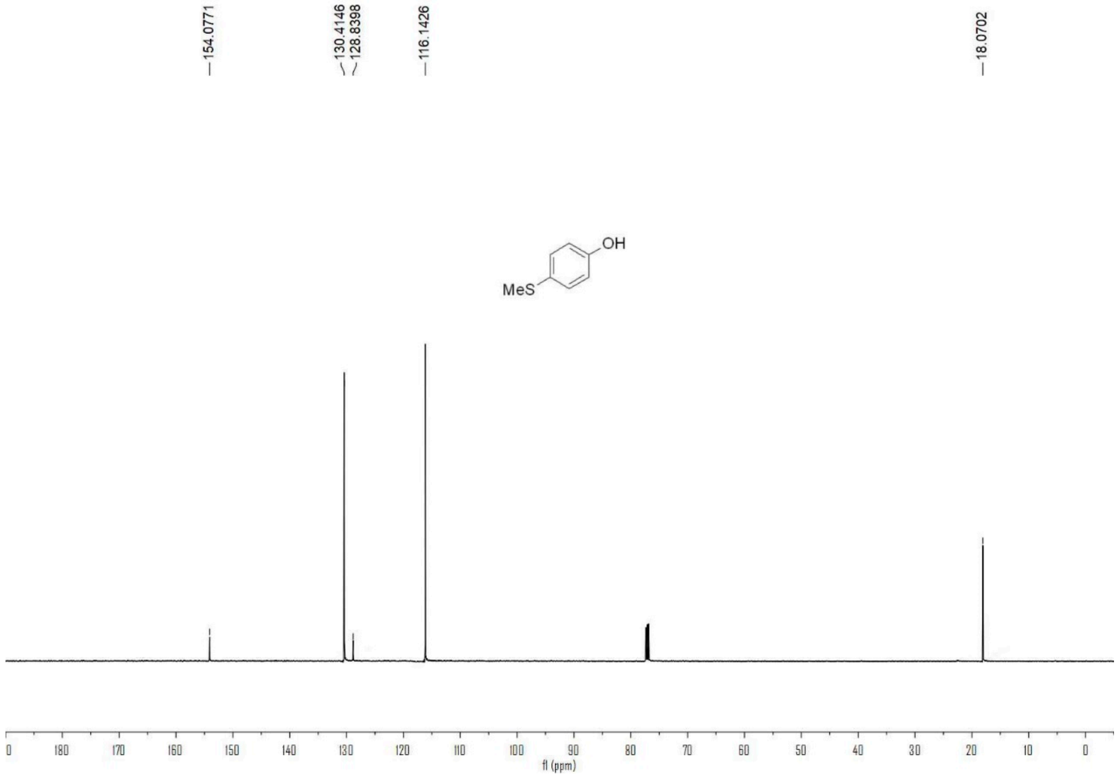

^{1}H NMR of 2g

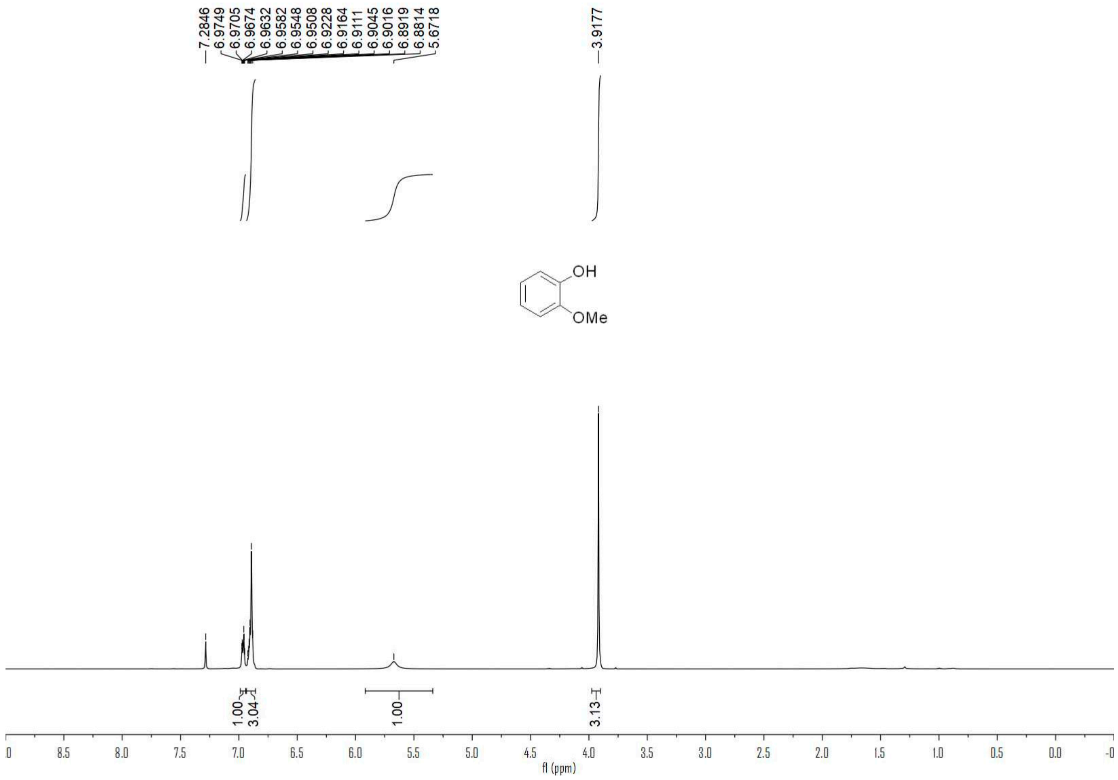

^{13}C NMR of 2g

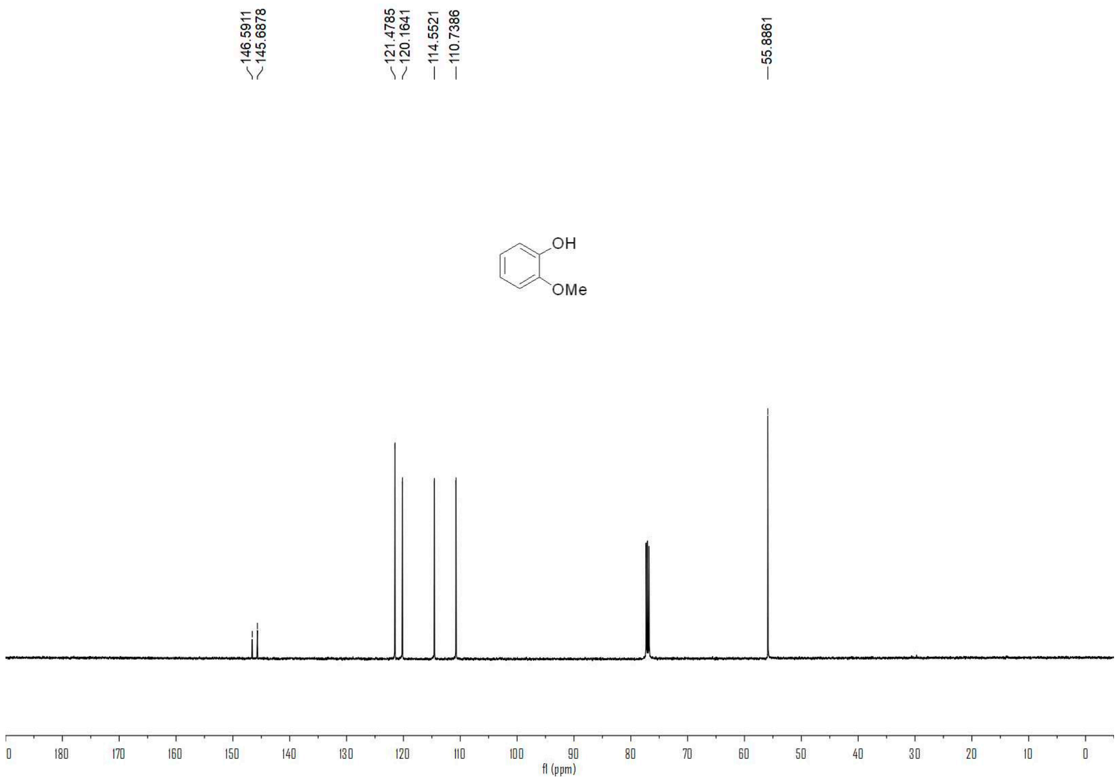

^{1}H NMR of 2h

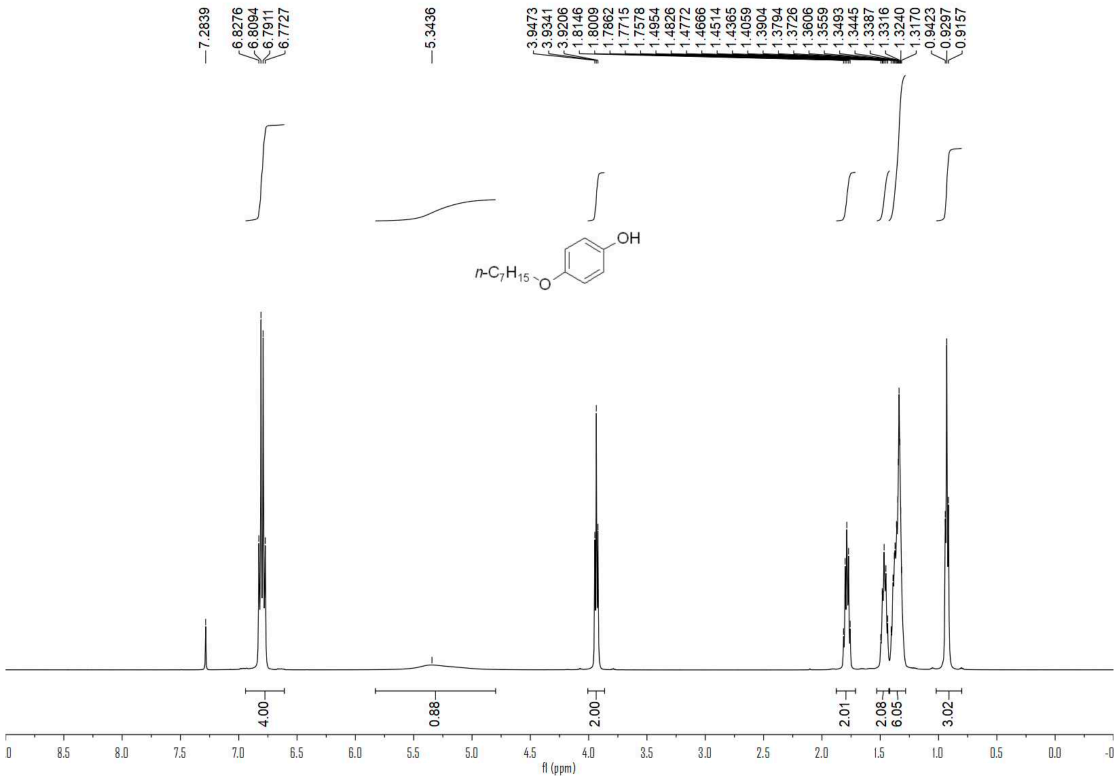

^{13}C NMR of 2h

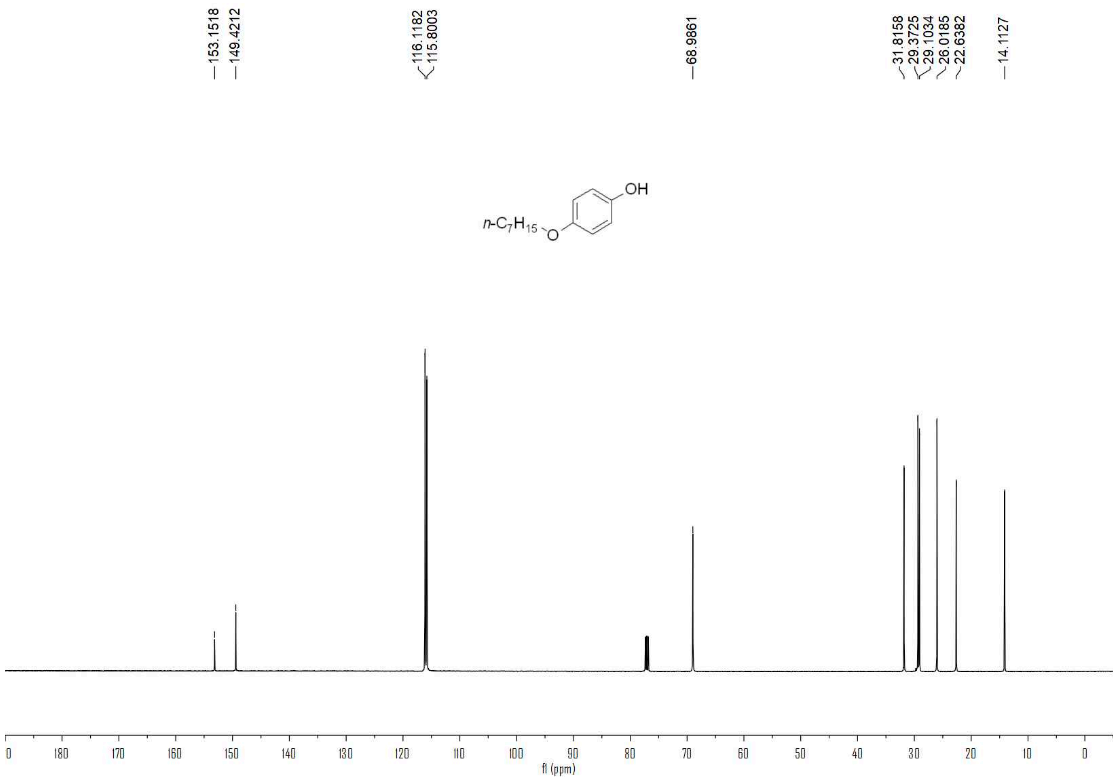

^{1}H NMR of 2i

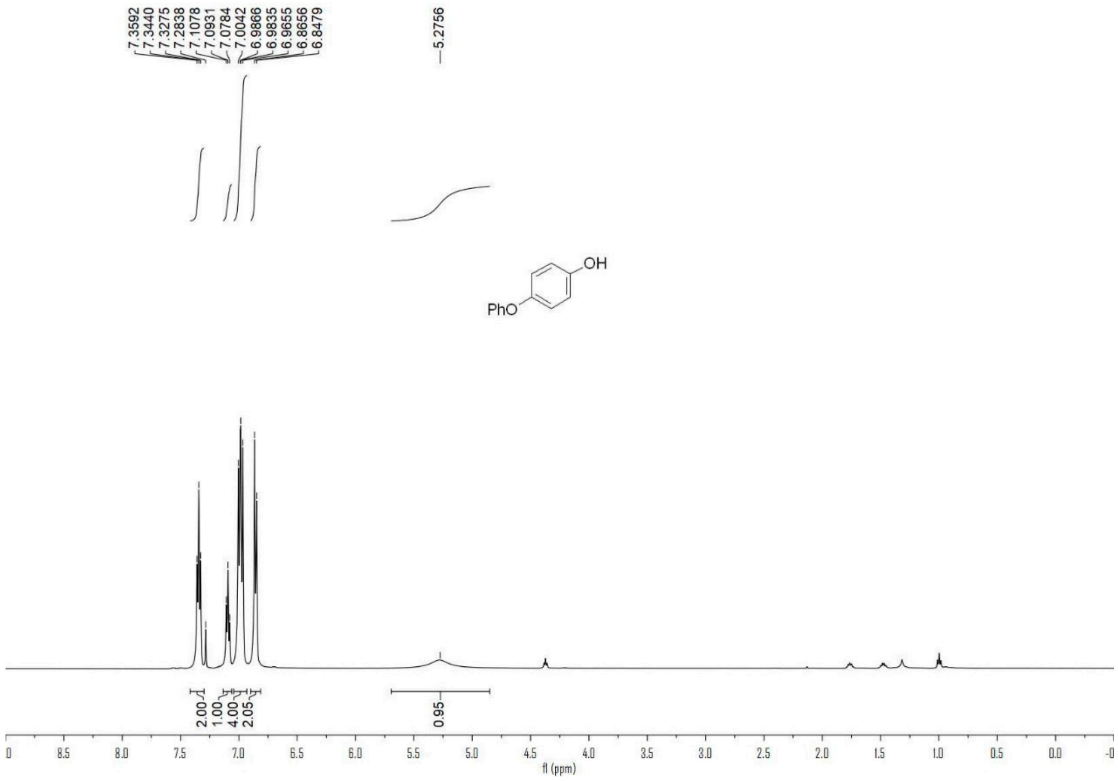

^{13}C NMR of 2i

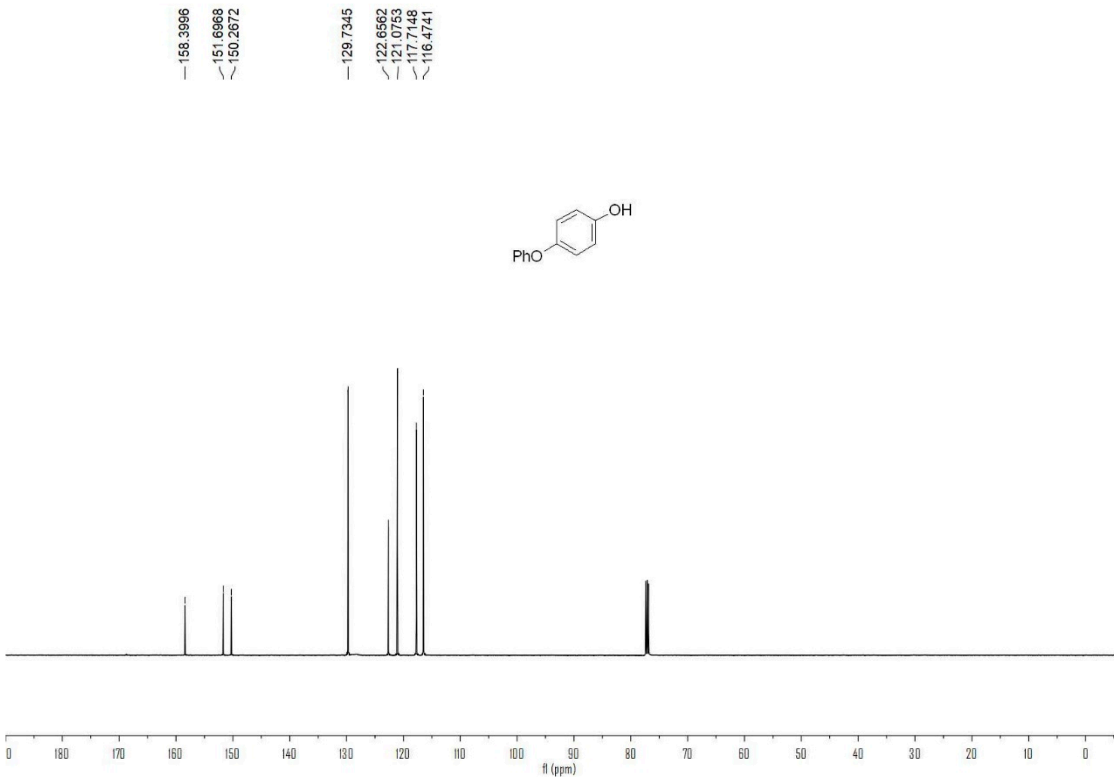

^{1}H NMR of 2j

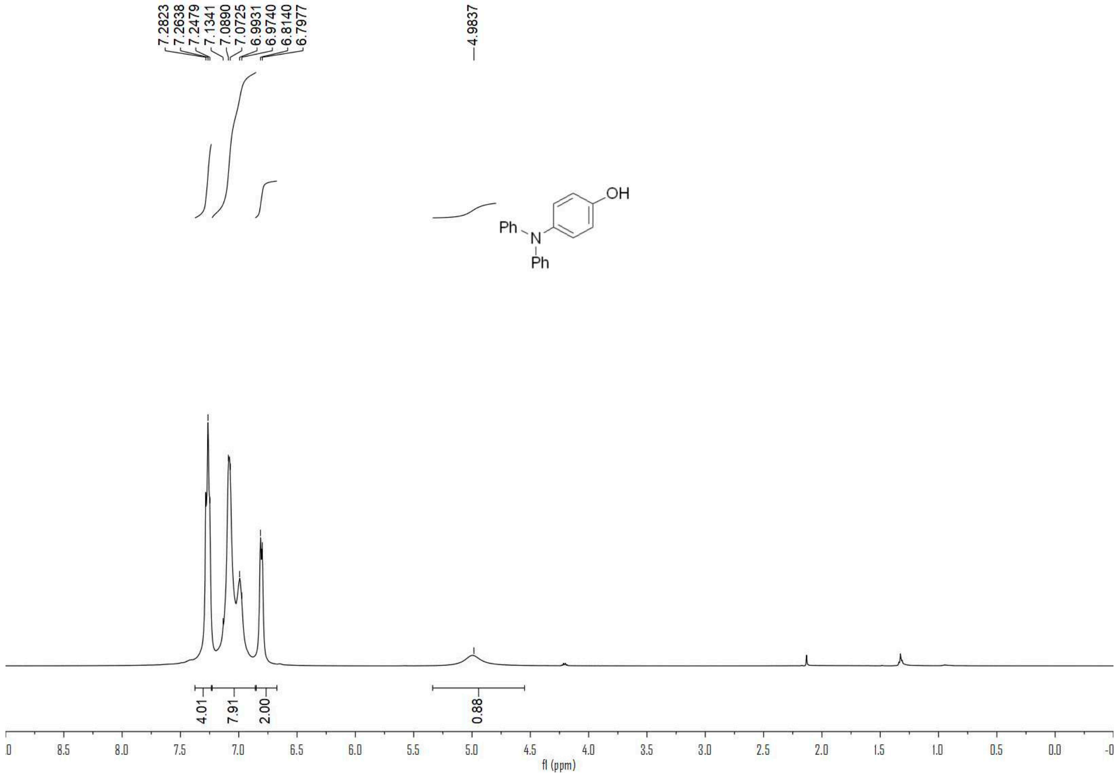

^{13}C NMR of 2j

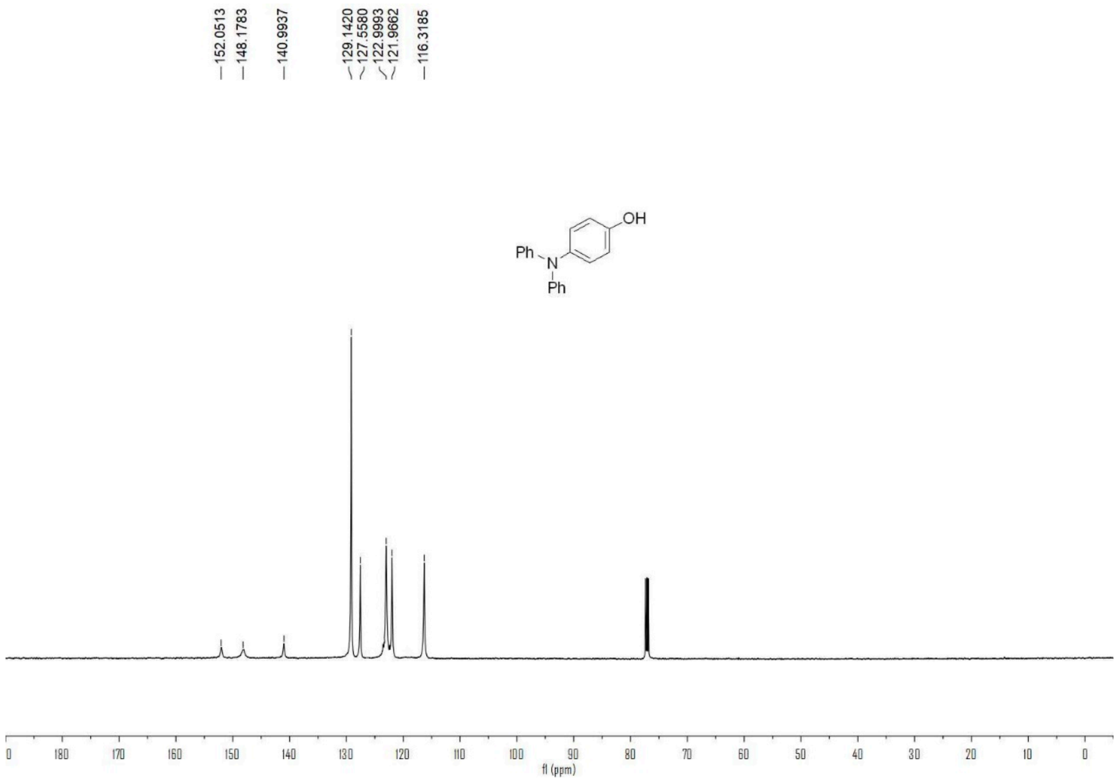

^{1}H NMR of 2k

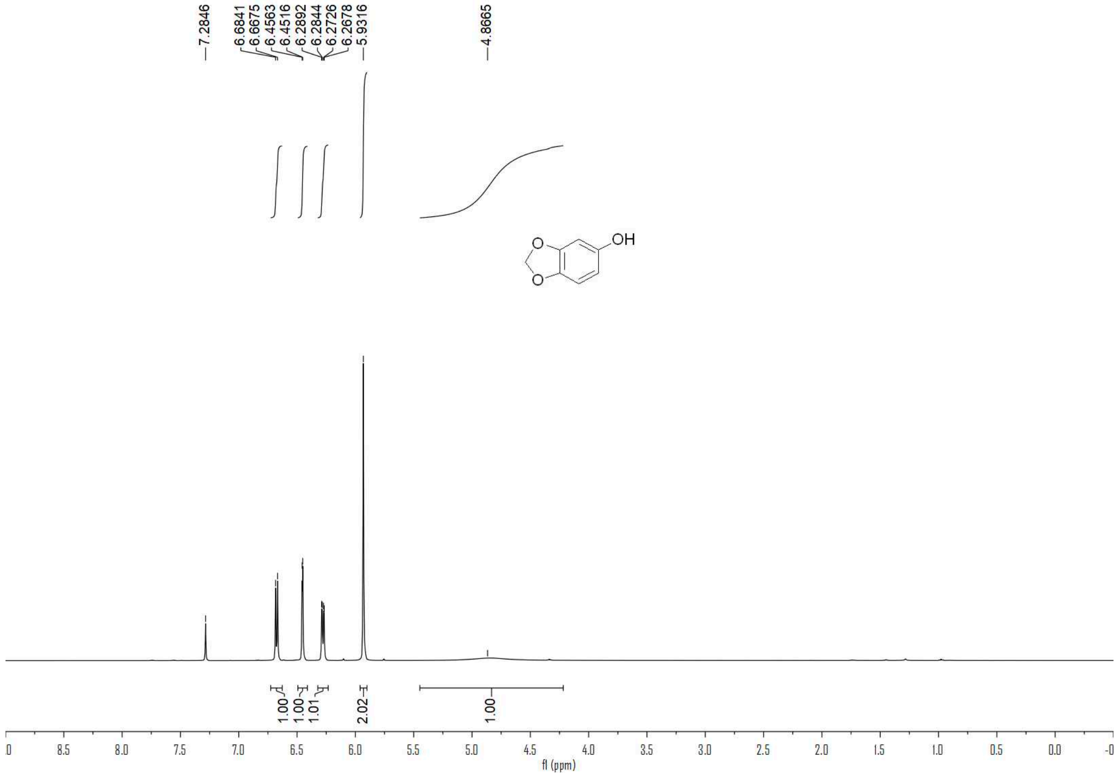

^{13}C NMR of 2k

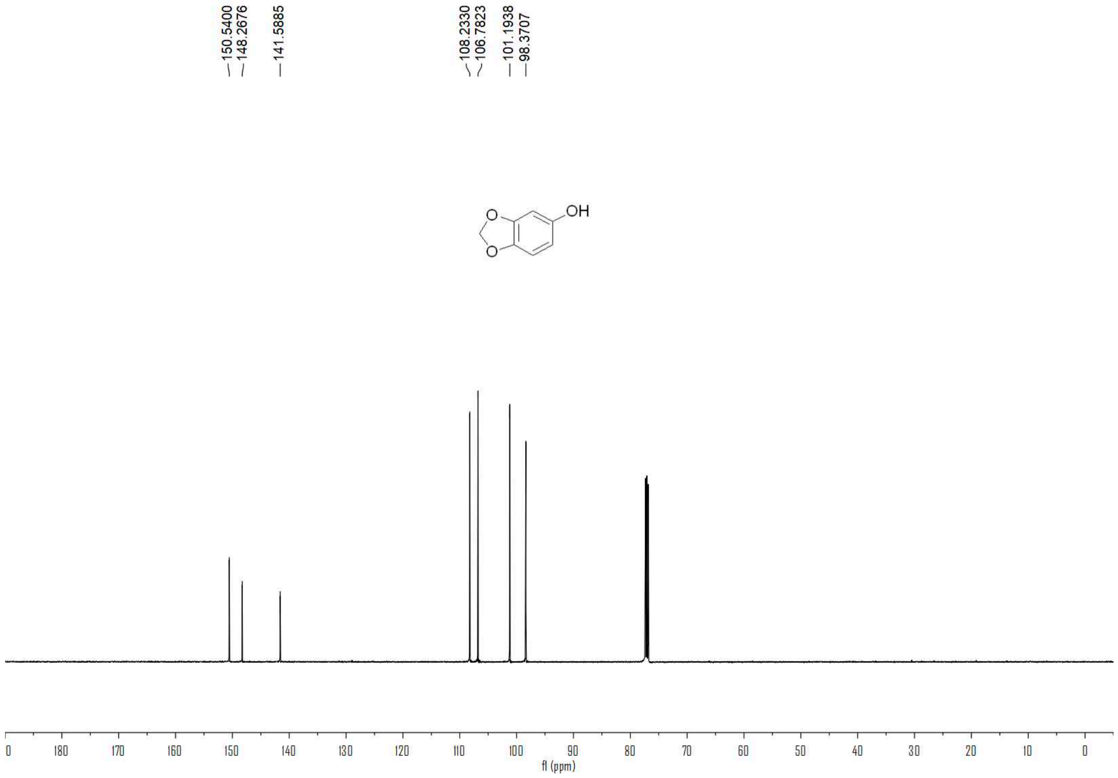

^{1}H NMR of 2l

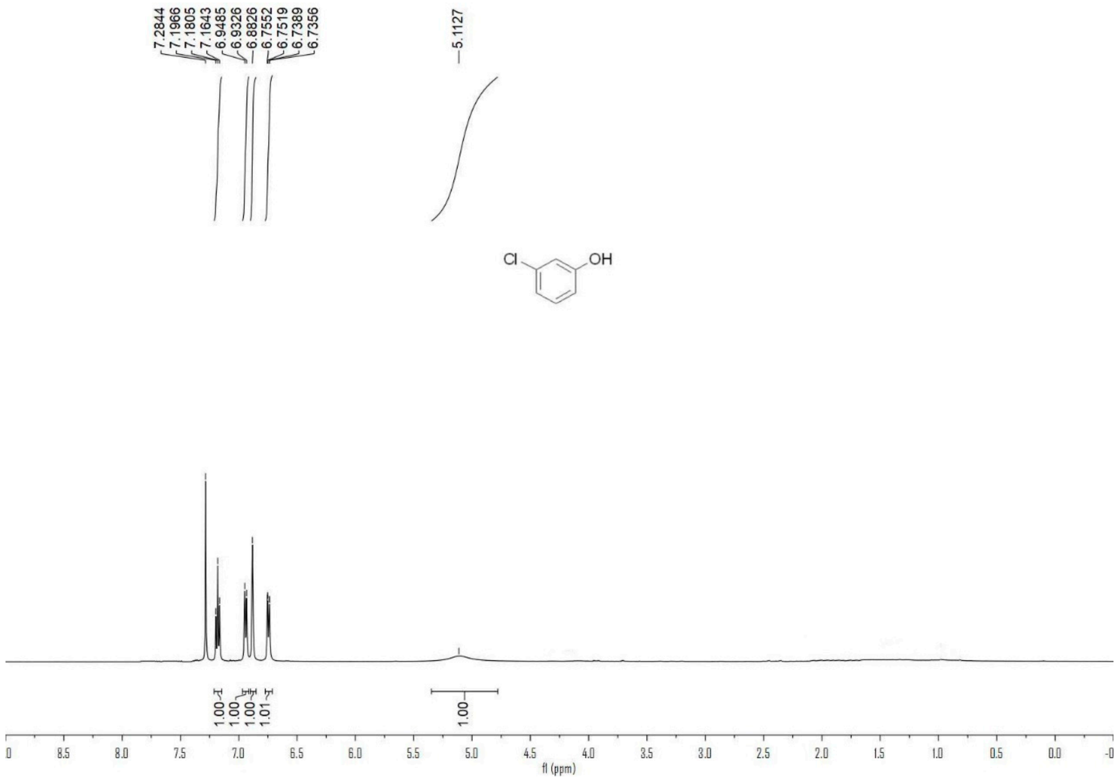

^{13}C NMR of 2l

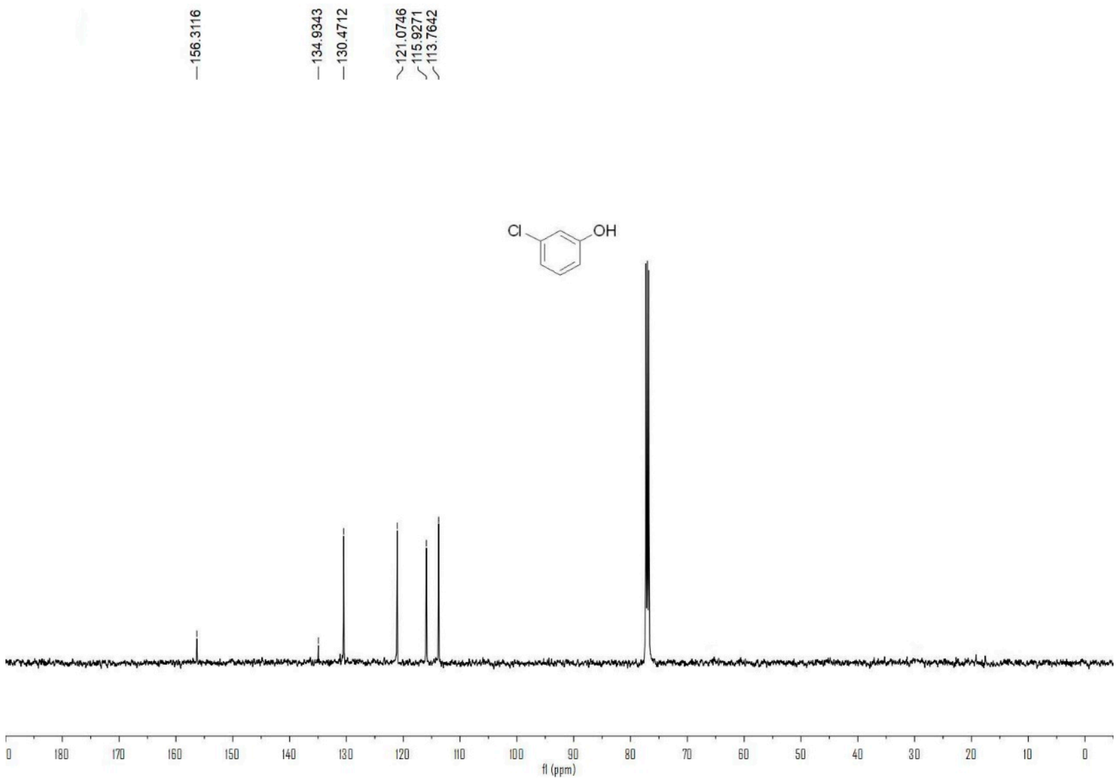

^{1}H NMR of 2m

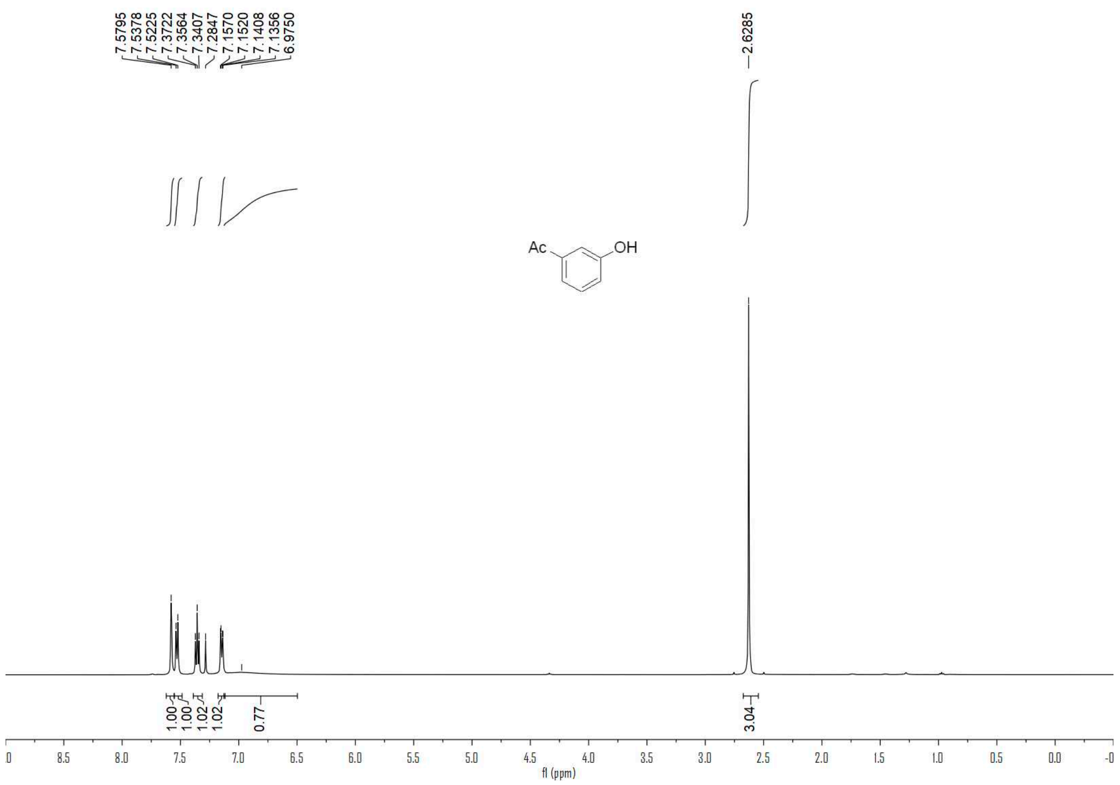

^{13}C NMR of 2m

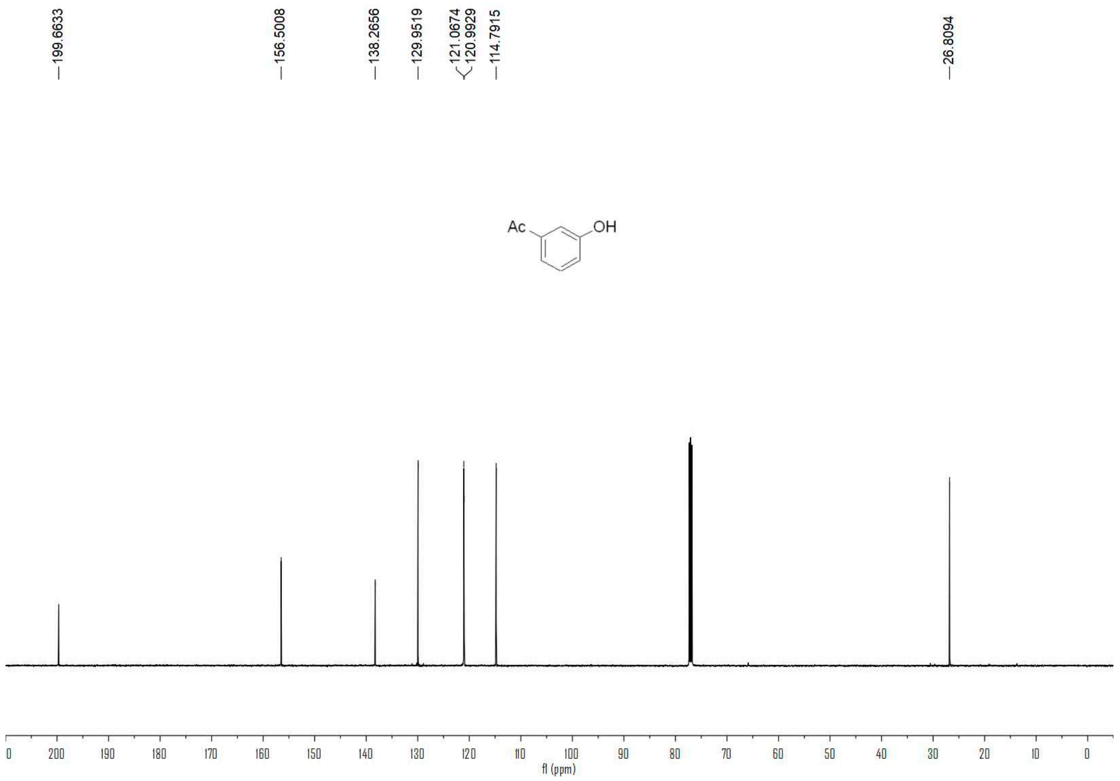

^{1}H NMR of 2n

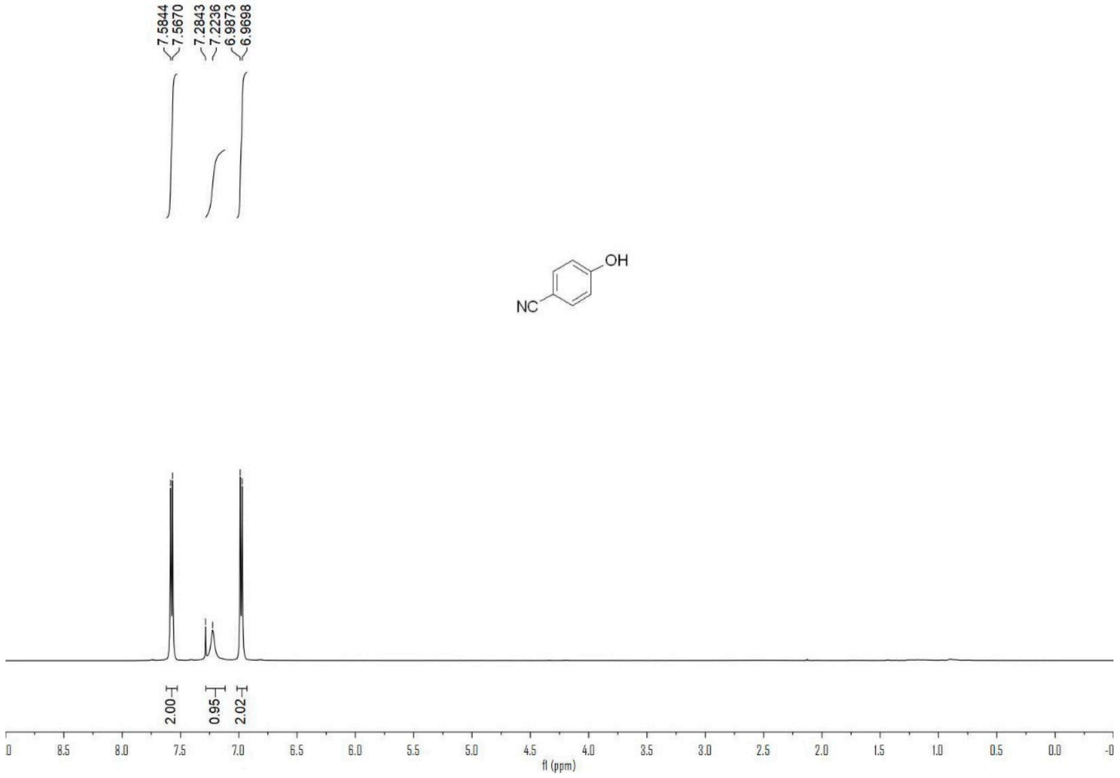

^{13}C NMR of 2n

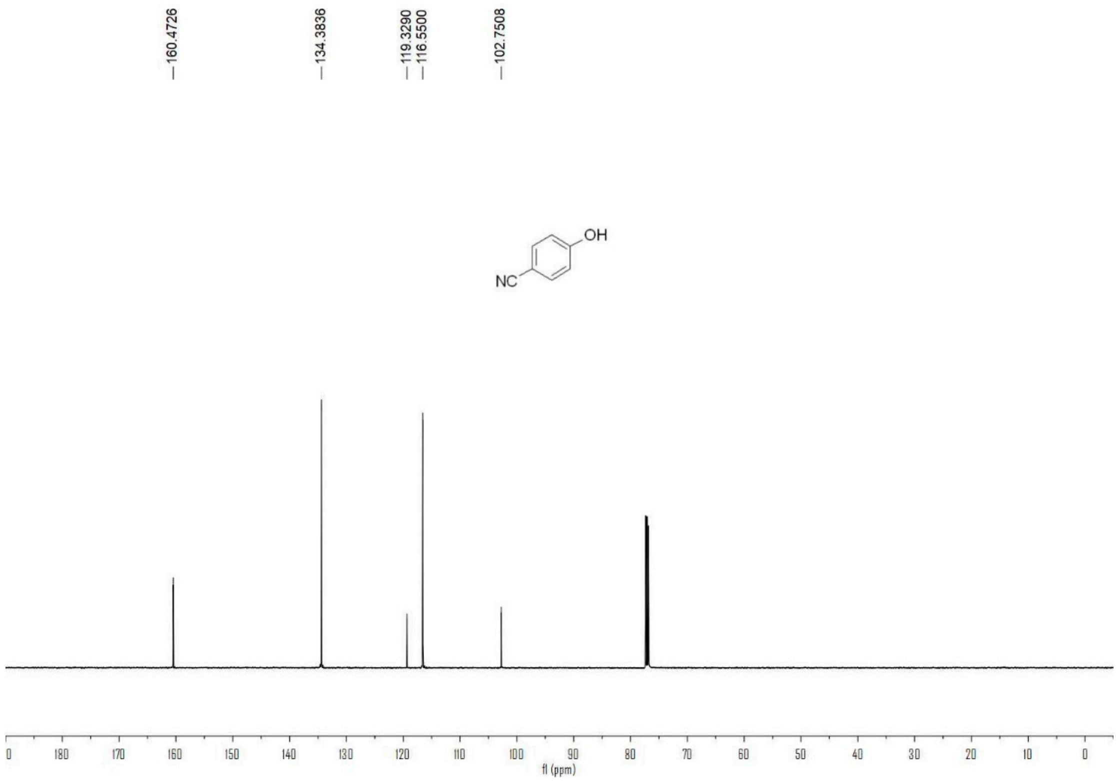

^{1}H NMR of 2o

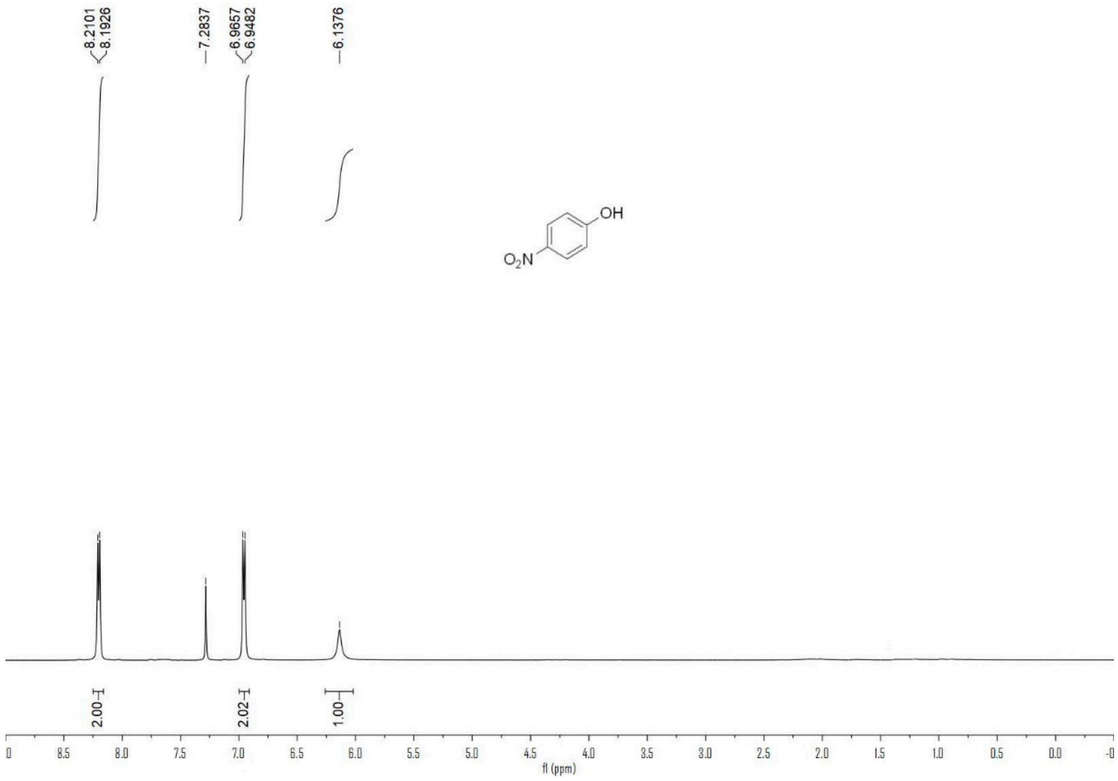

^{13}C NMR of 2o

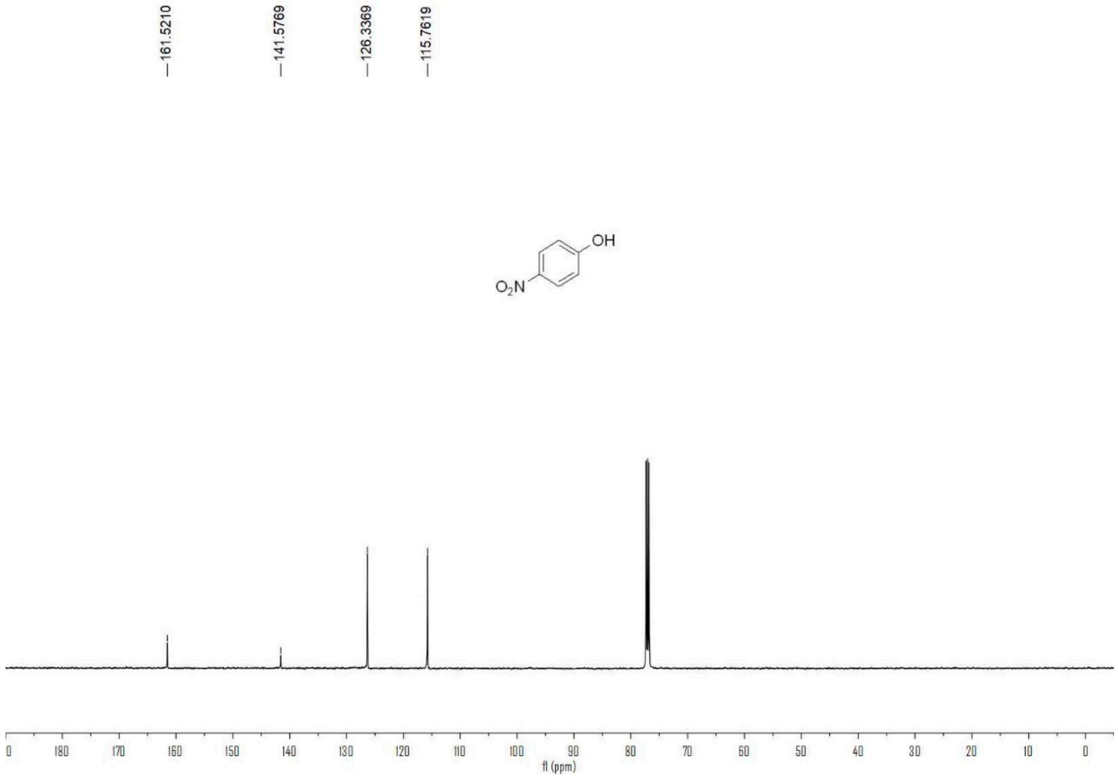

^{1}H NMR of 2p

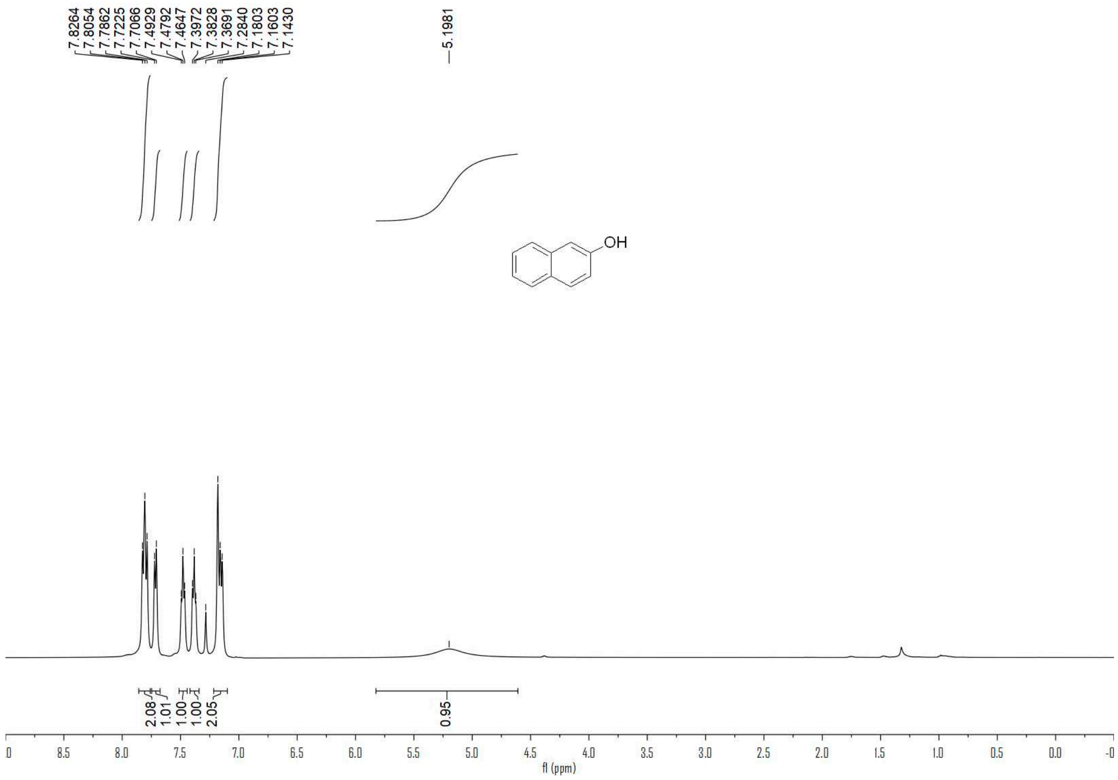

^{13}C NMR of 2p

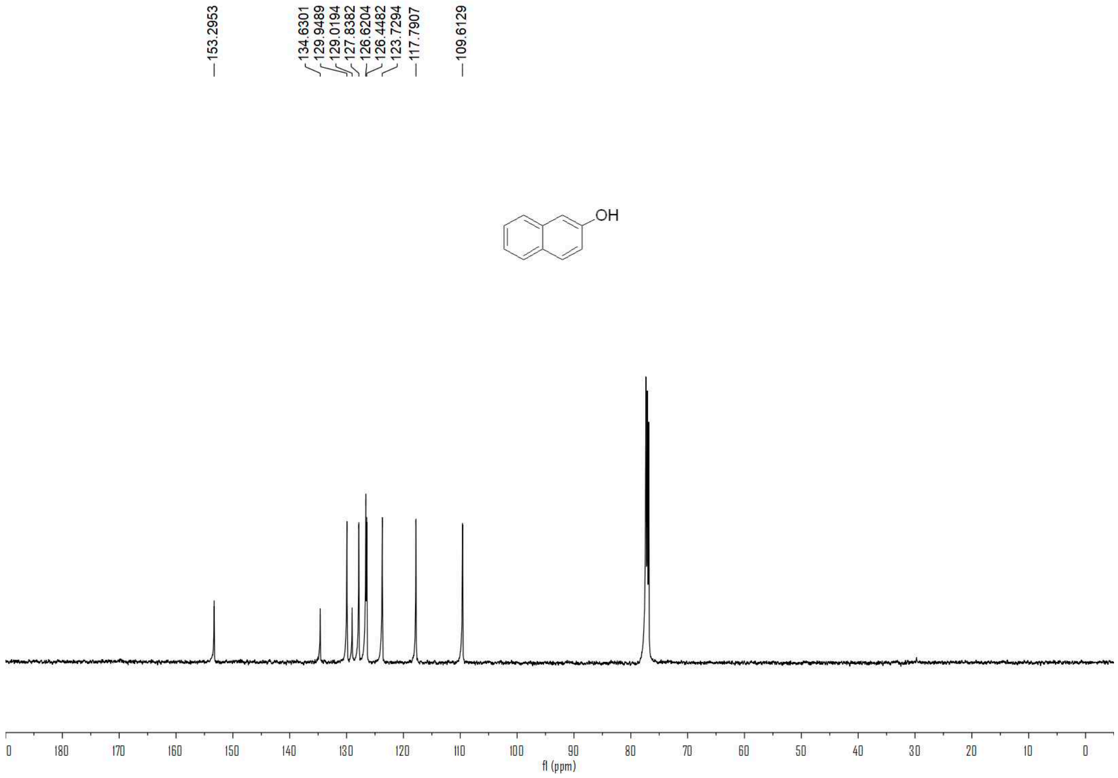

^{1}H NMR of 2q

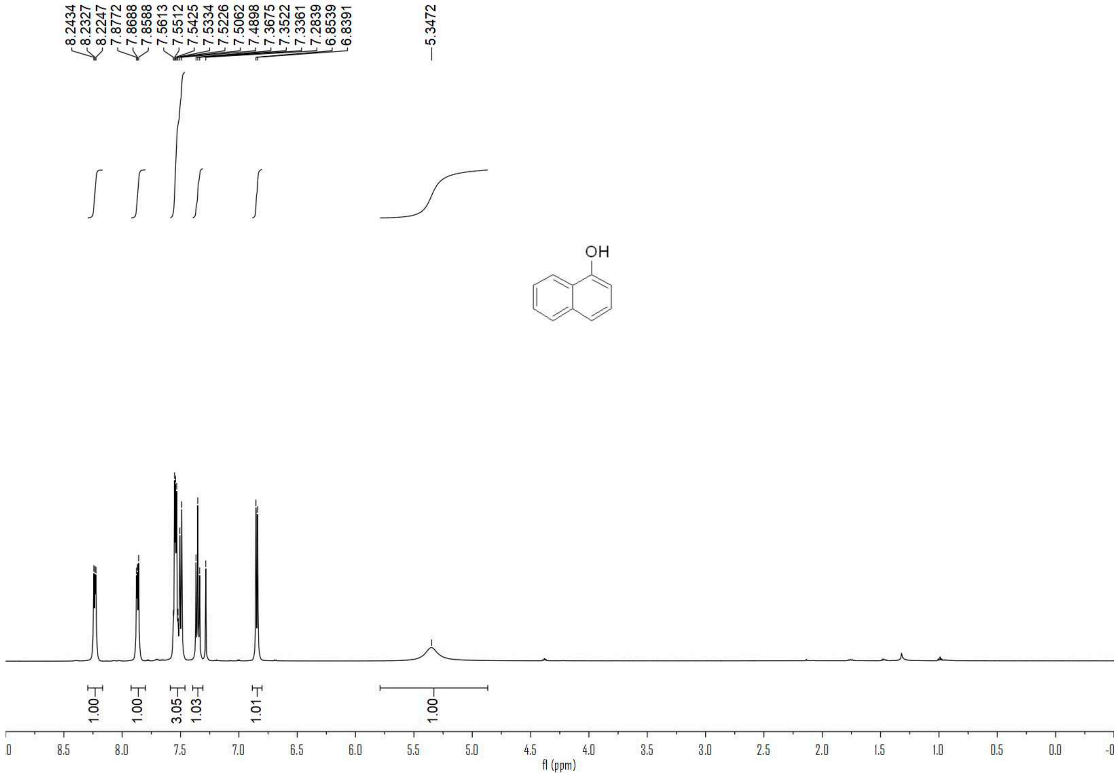

^{13}C NMR of 2q

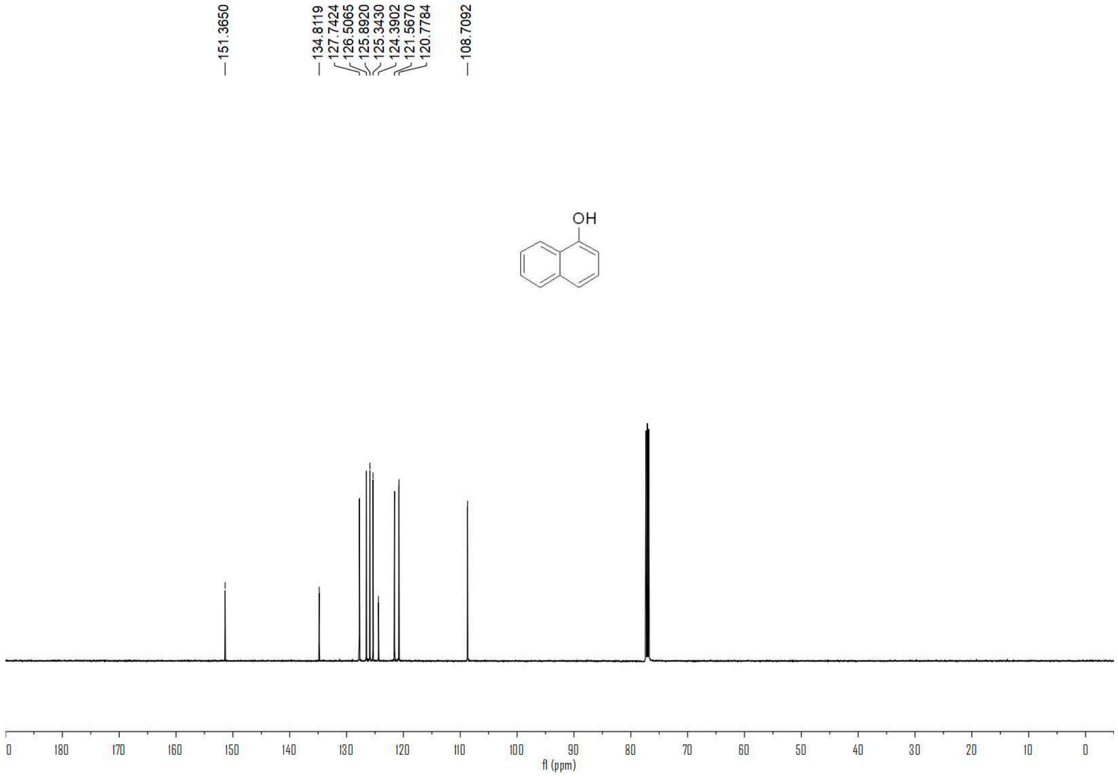

^1H NMR of 2r

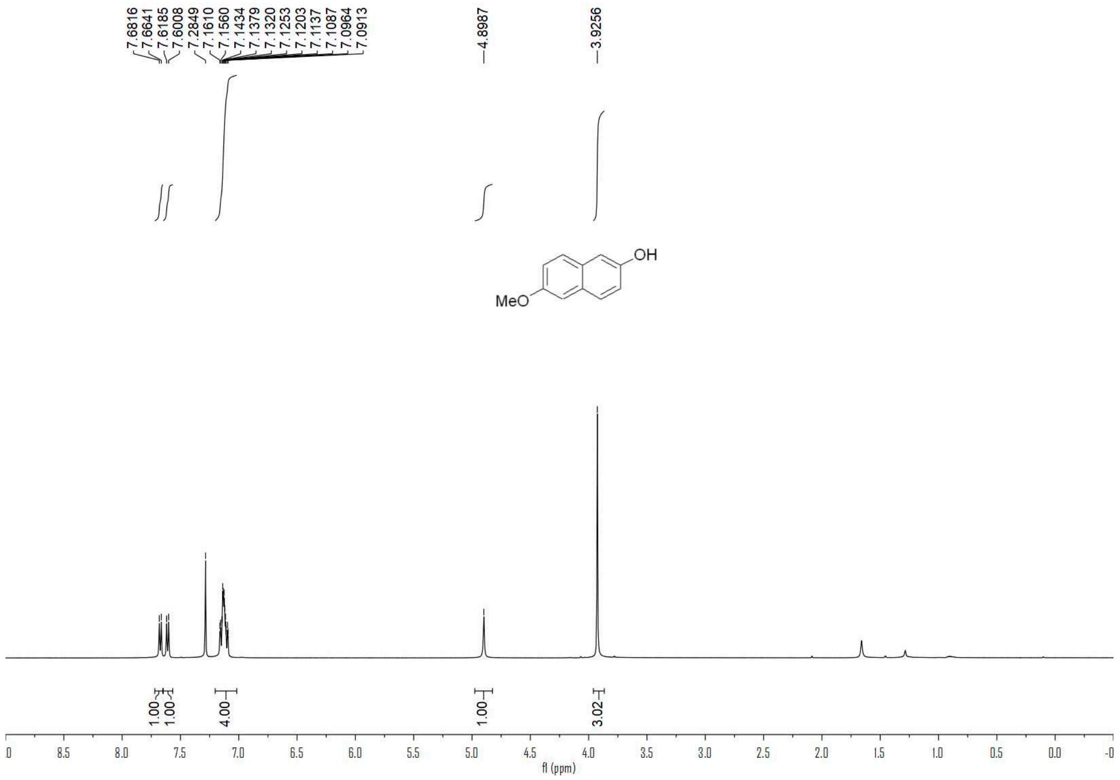

^{13}C NMR of 2r

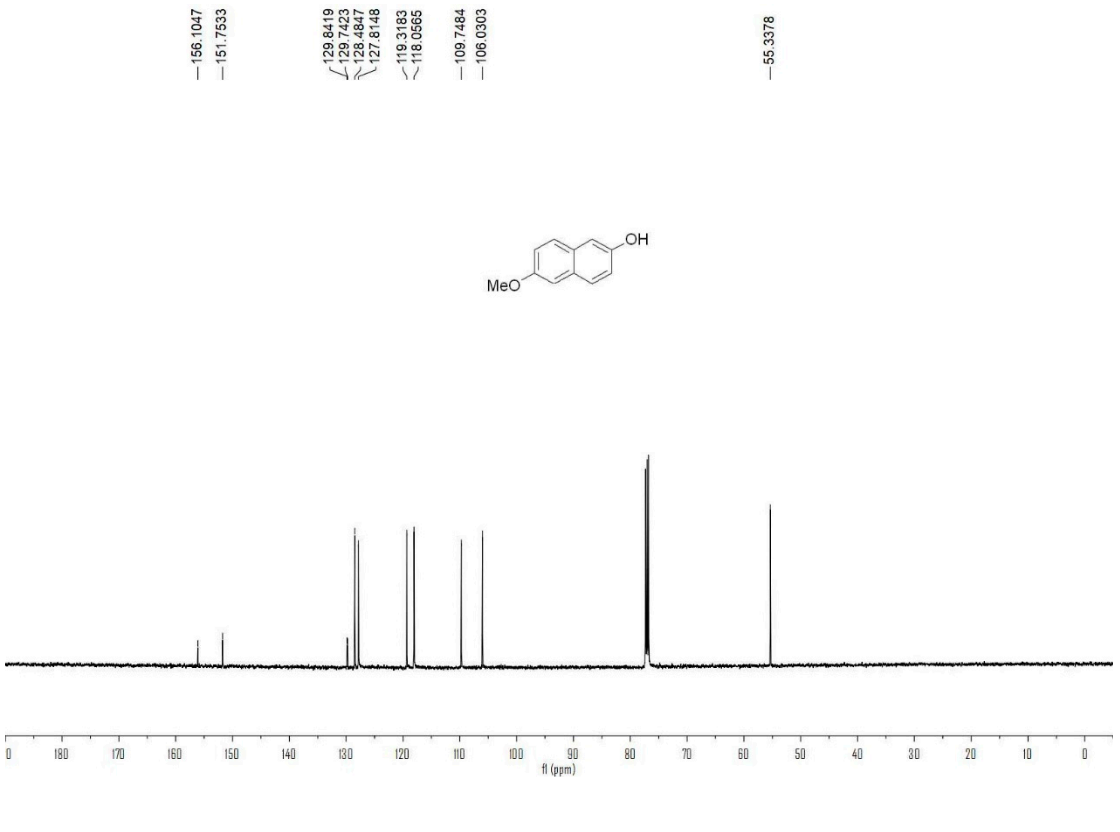

^{1}H NMR of 2s

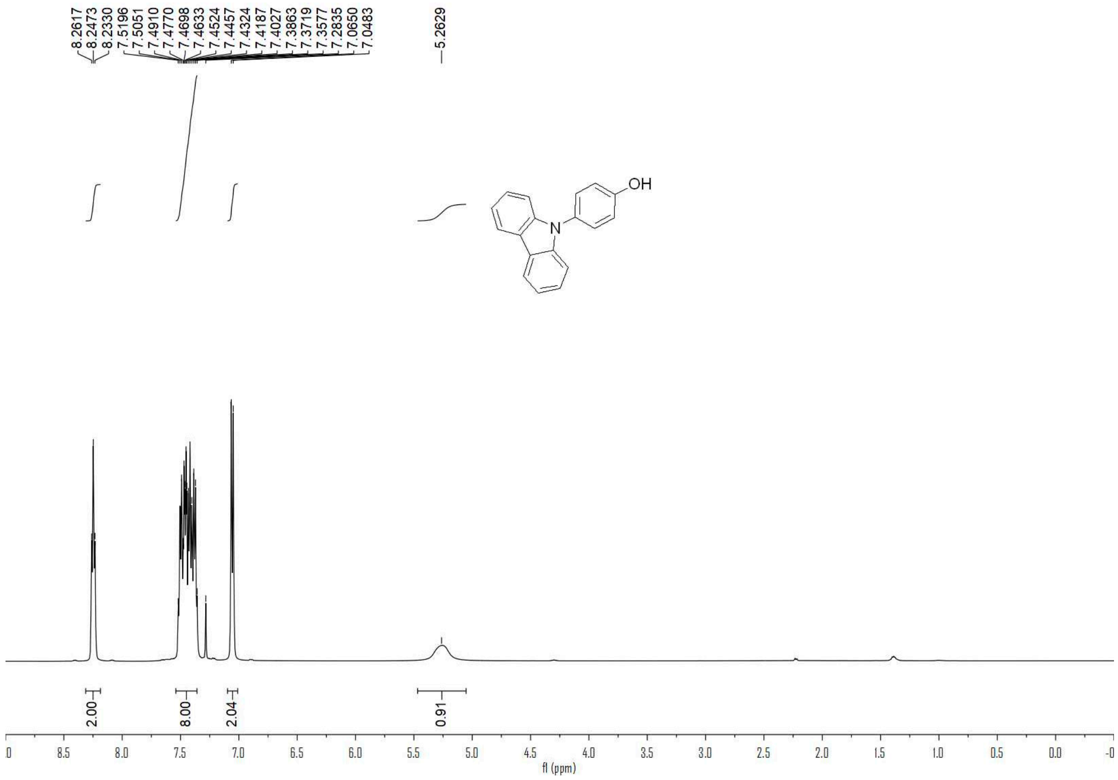

^{13}C NMR of 2s

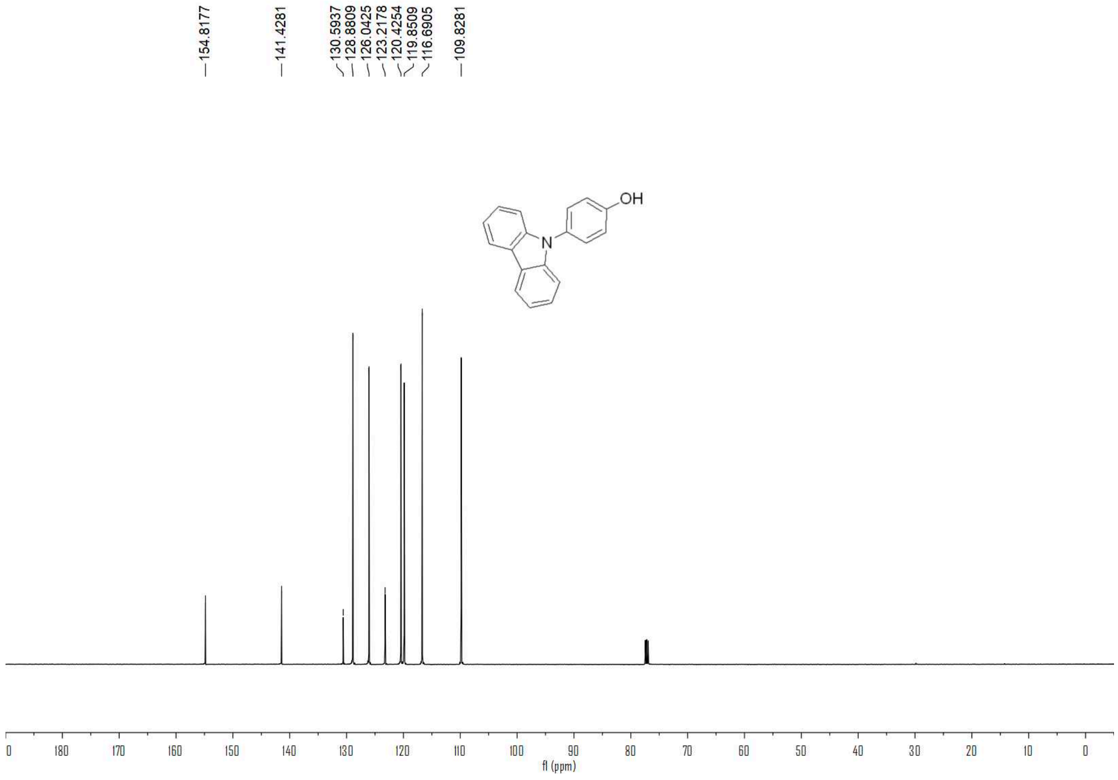

^{1}H NMR of 2t

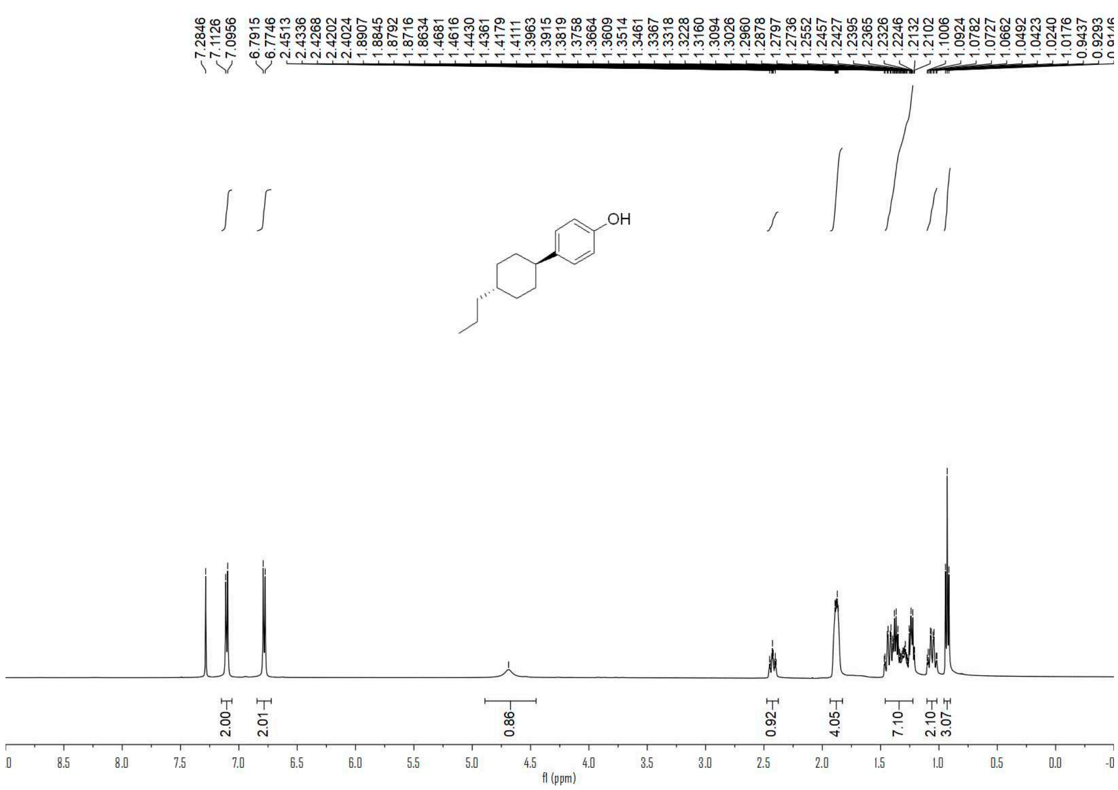

^{13}C NMR of 2t

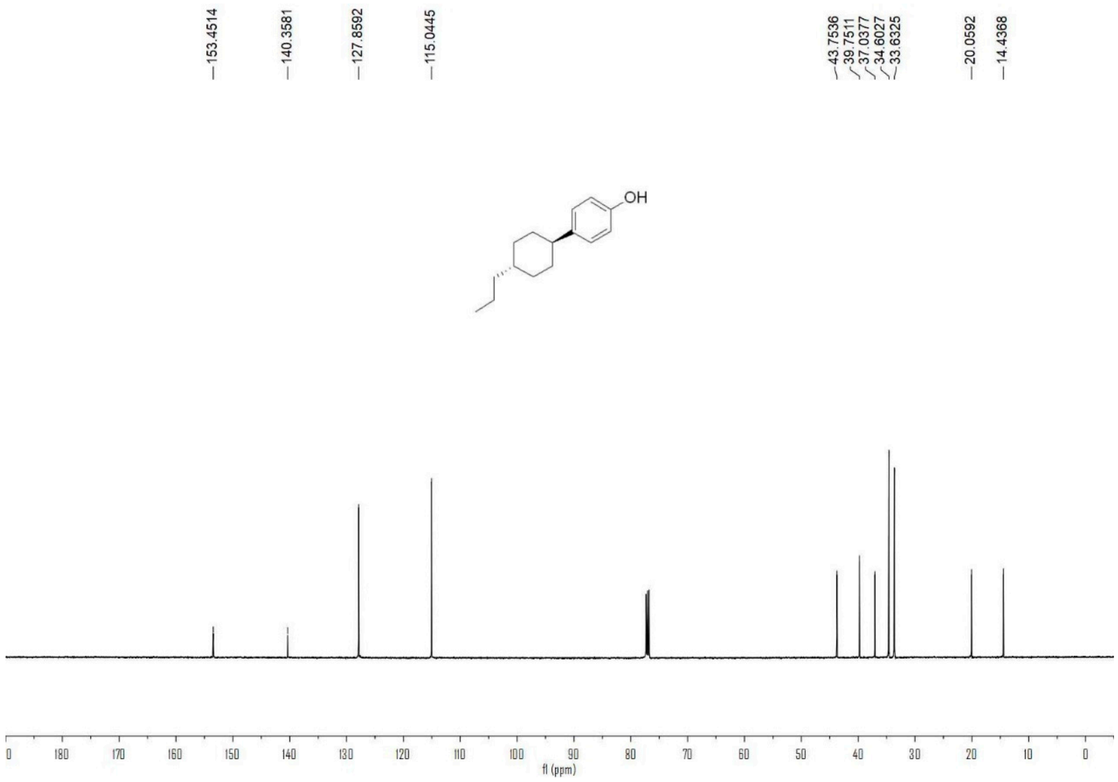

^{1}H NMR of 4a

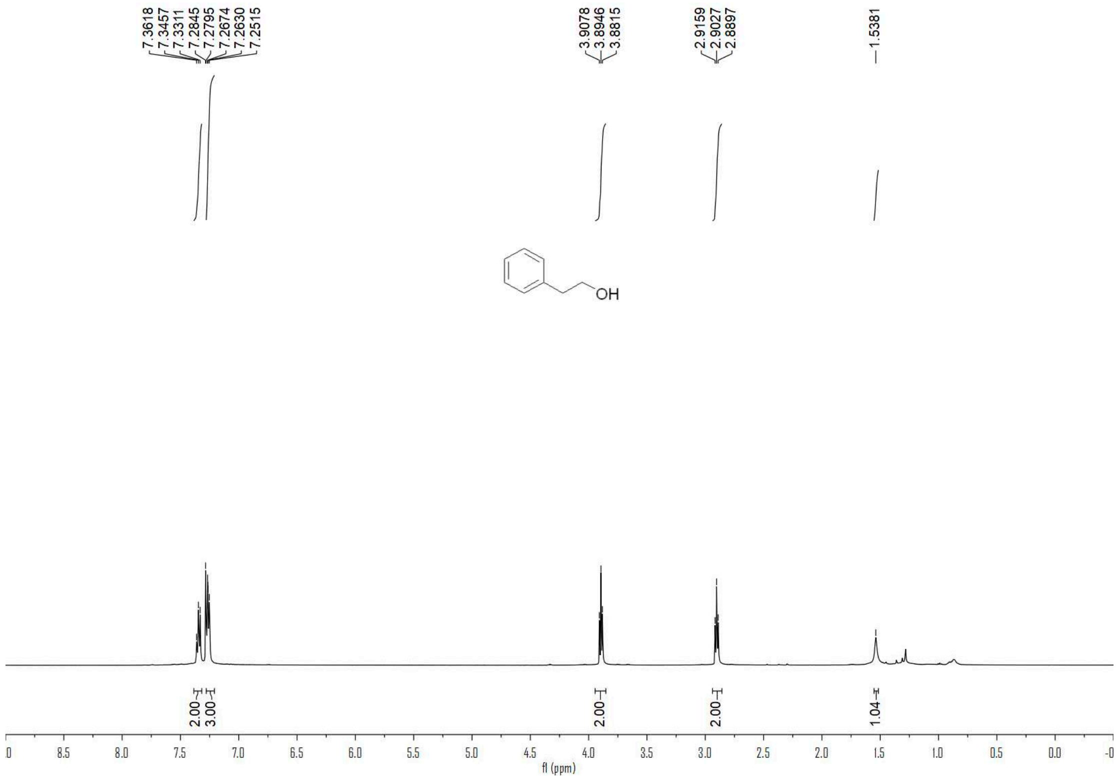

^{13}C NMR of 4a

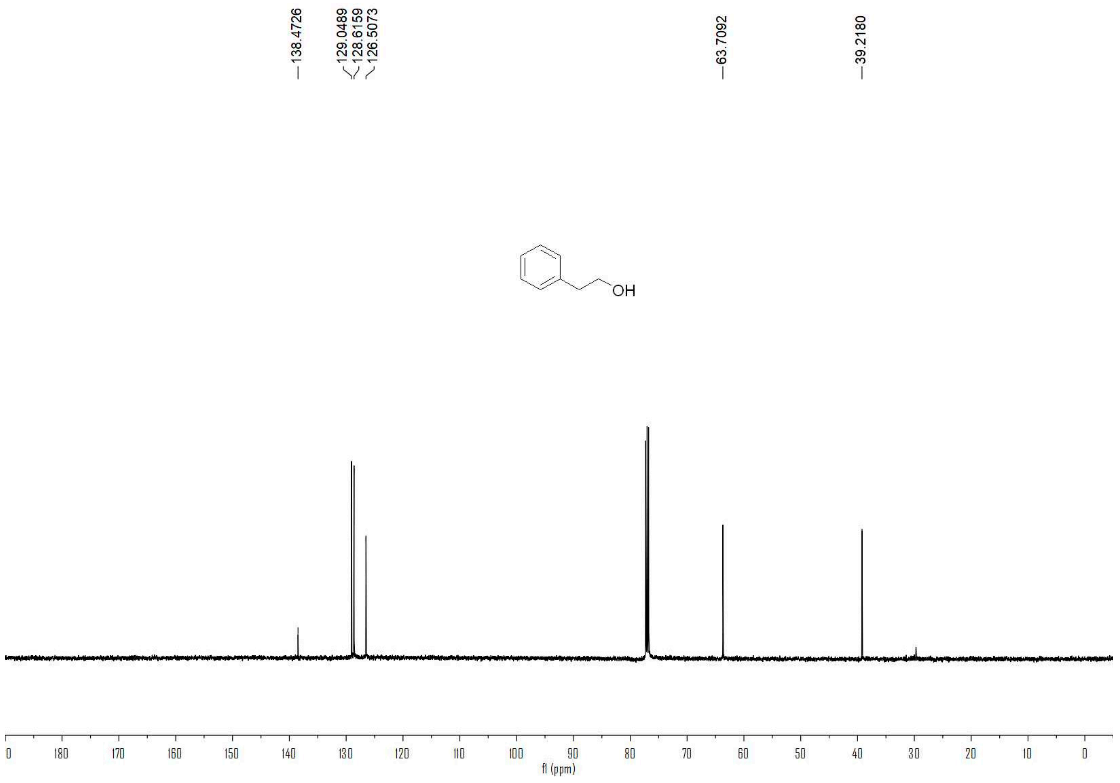

^{1}H NMR of 4b

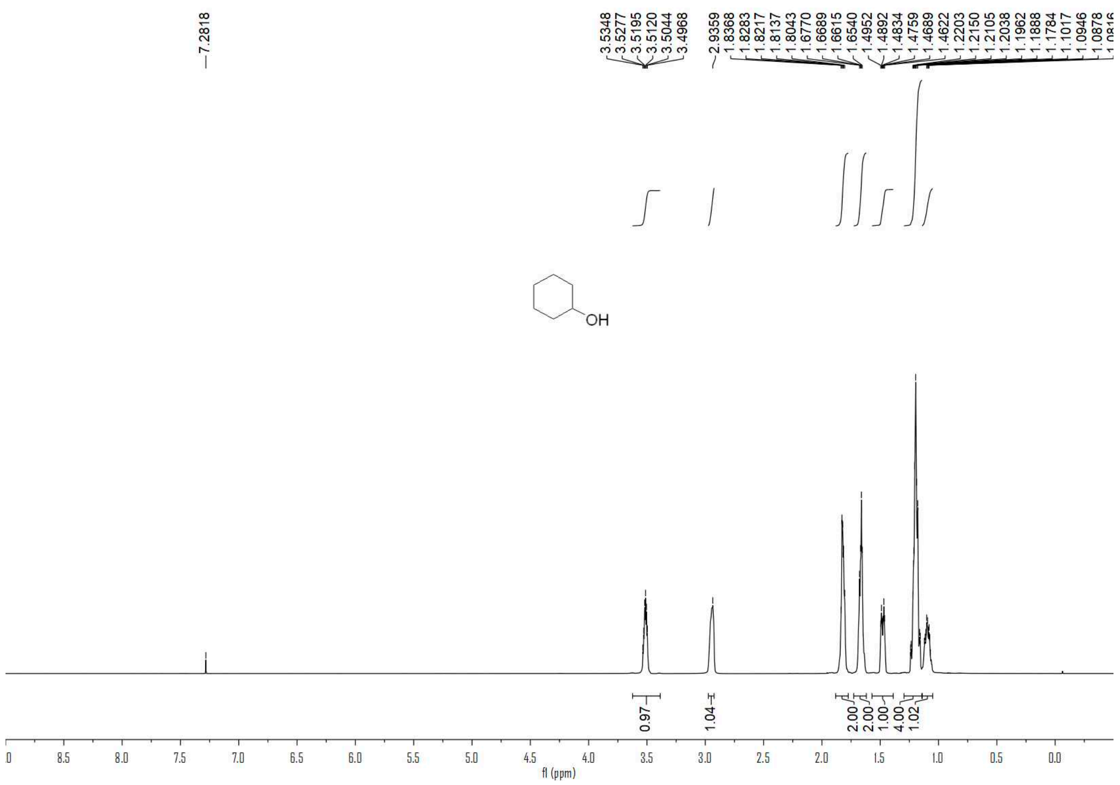

^{13}C NMR of 4b

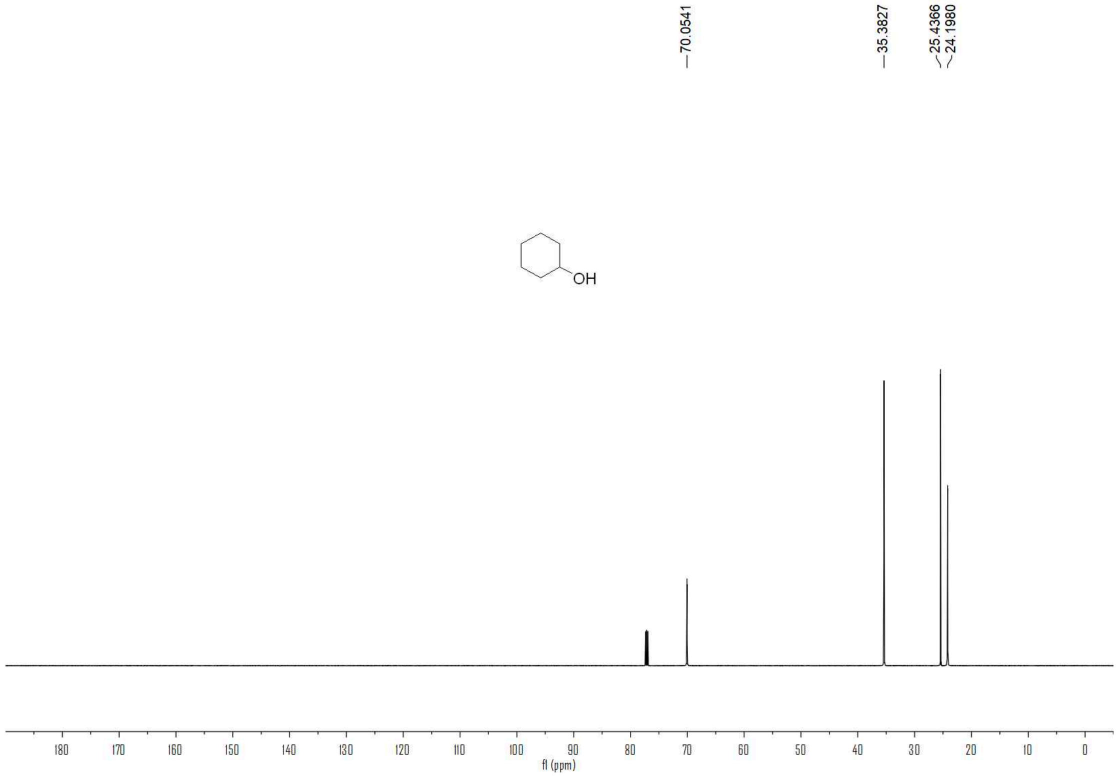

^{1}H NMR of 4c

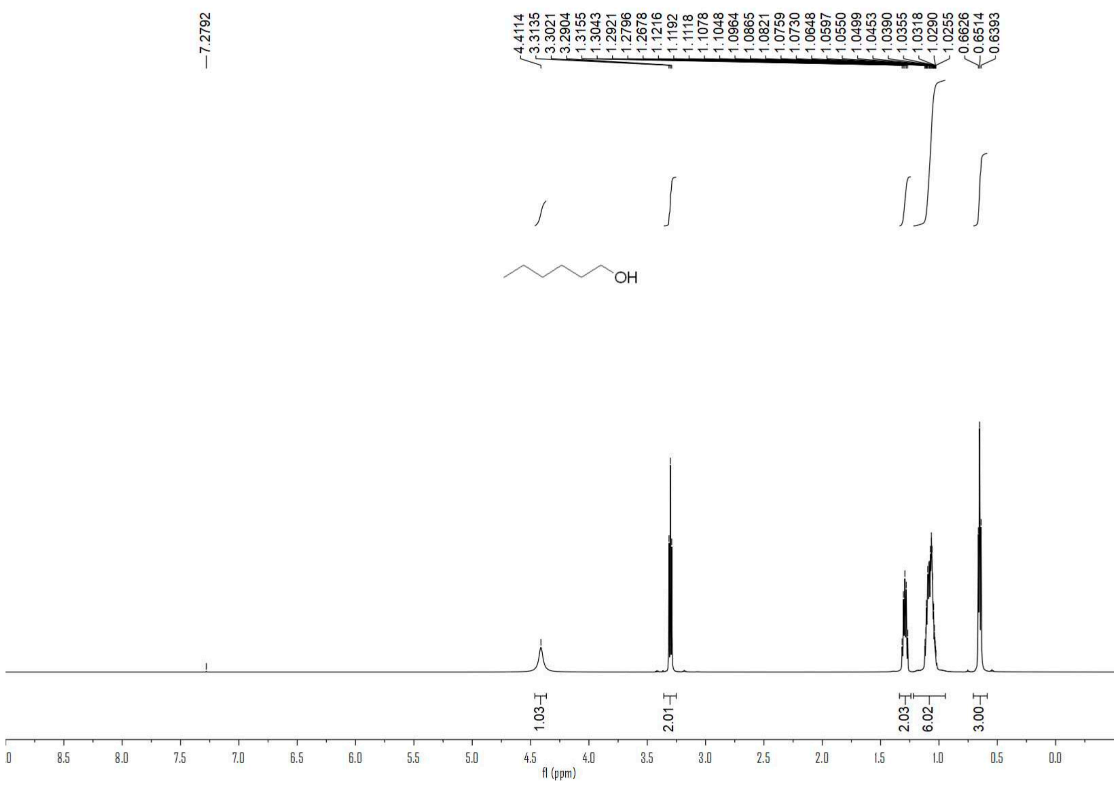

^{13}C NMR of 4c

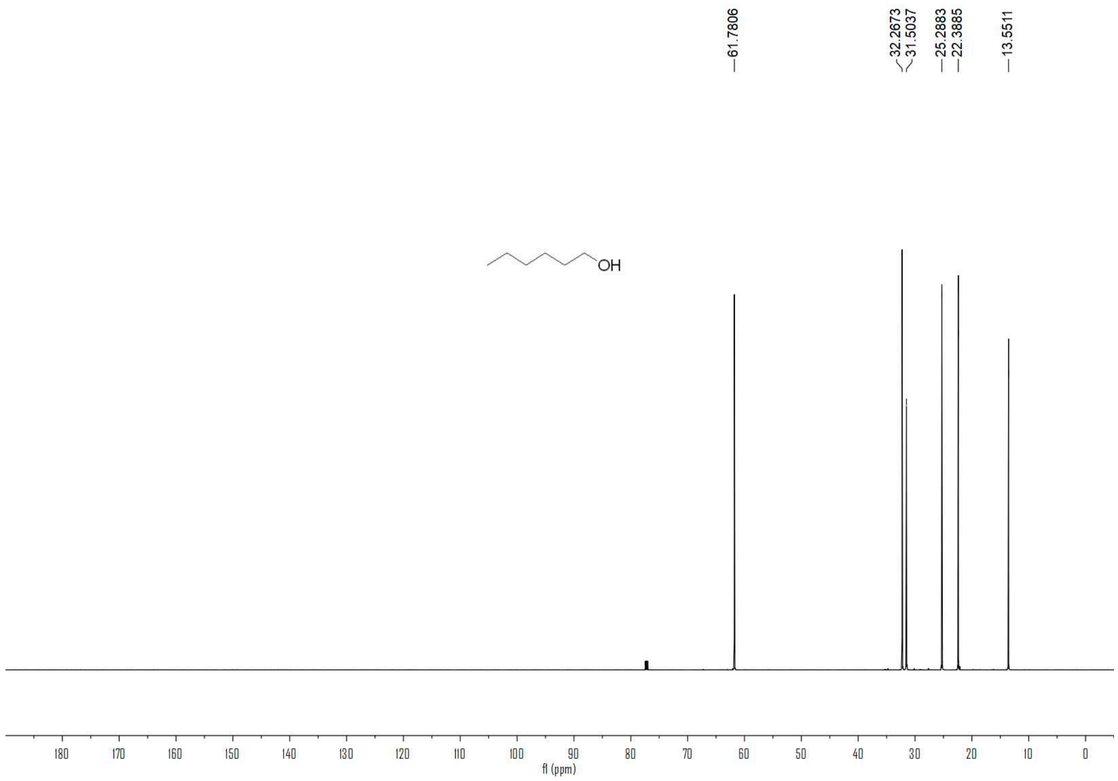

^{1}H NMR of 4d

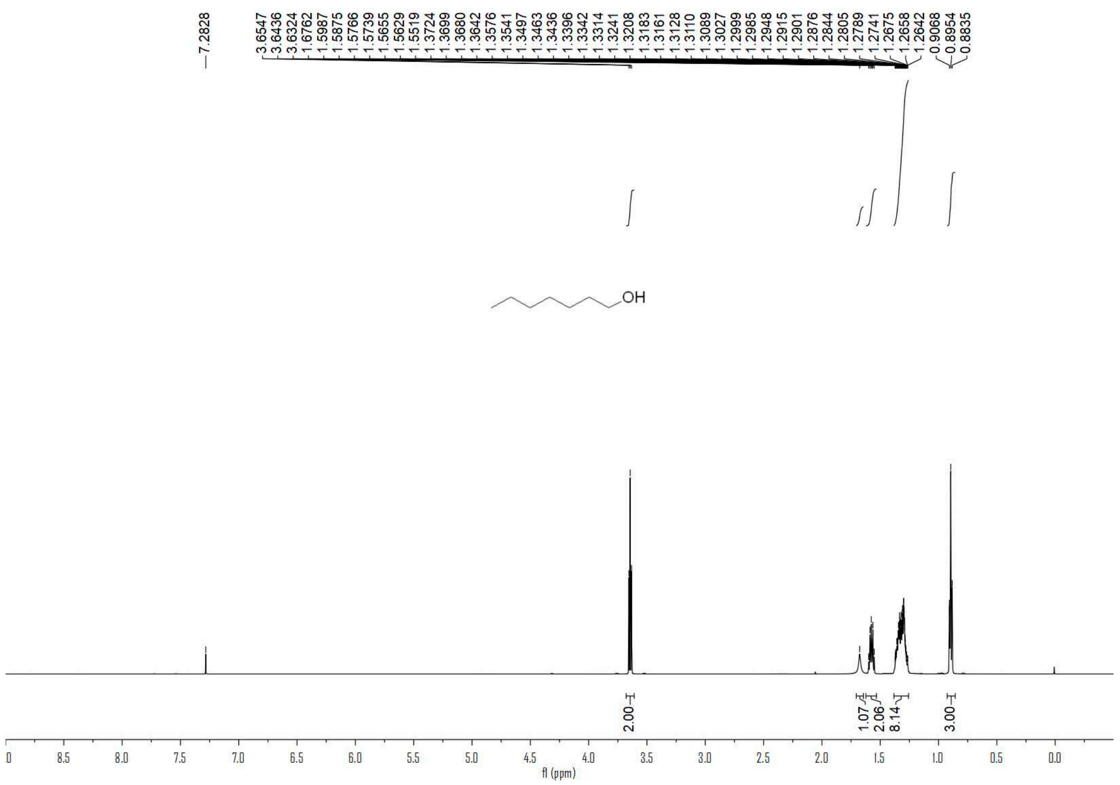

^{13}C NMR of 4d

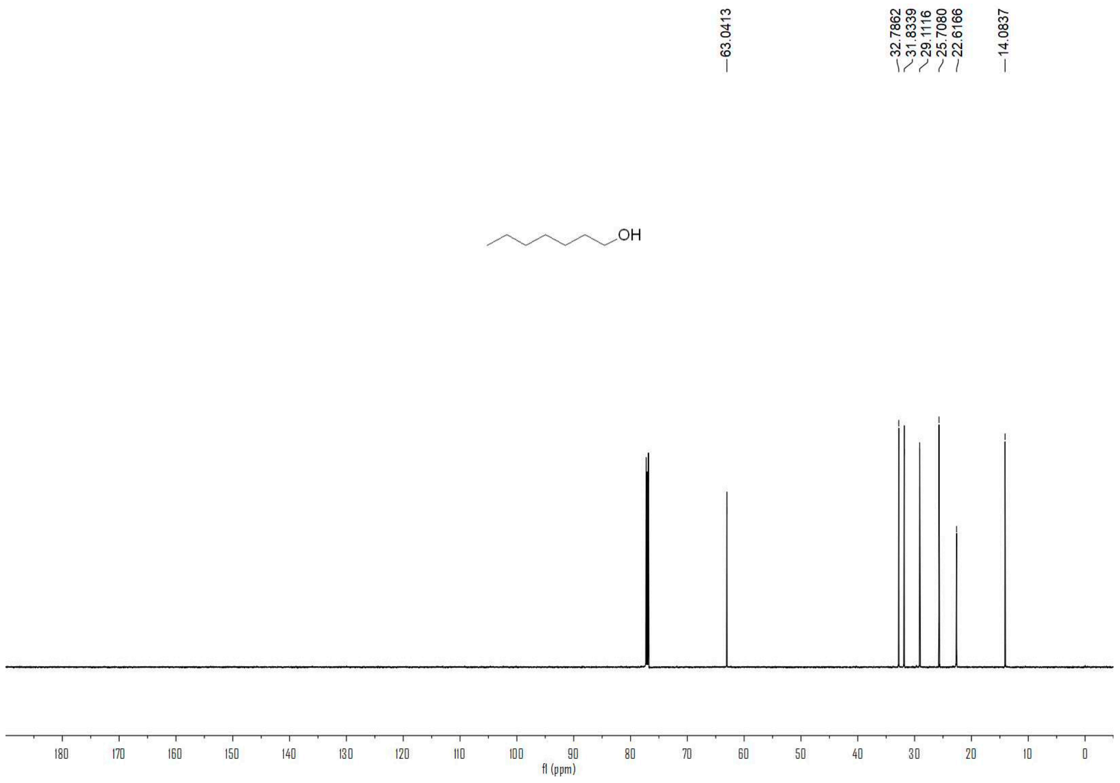

^{1}H NMR of 4e

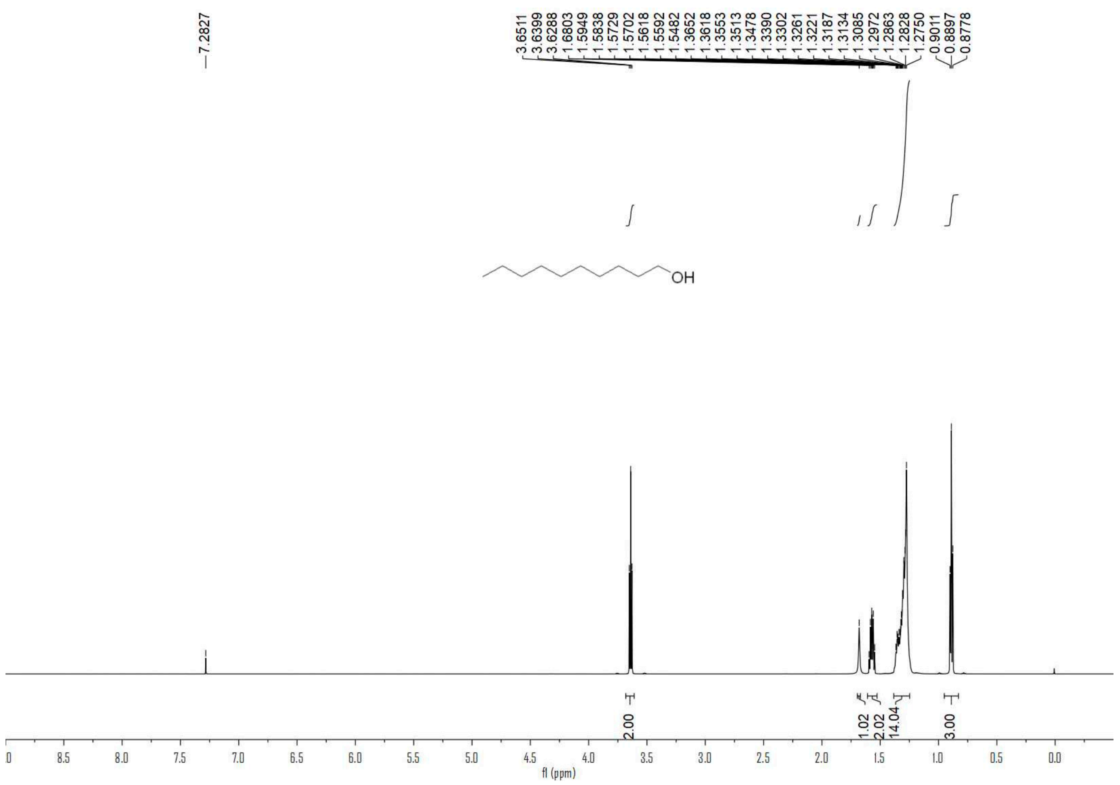

^{13}C NMR of 4e

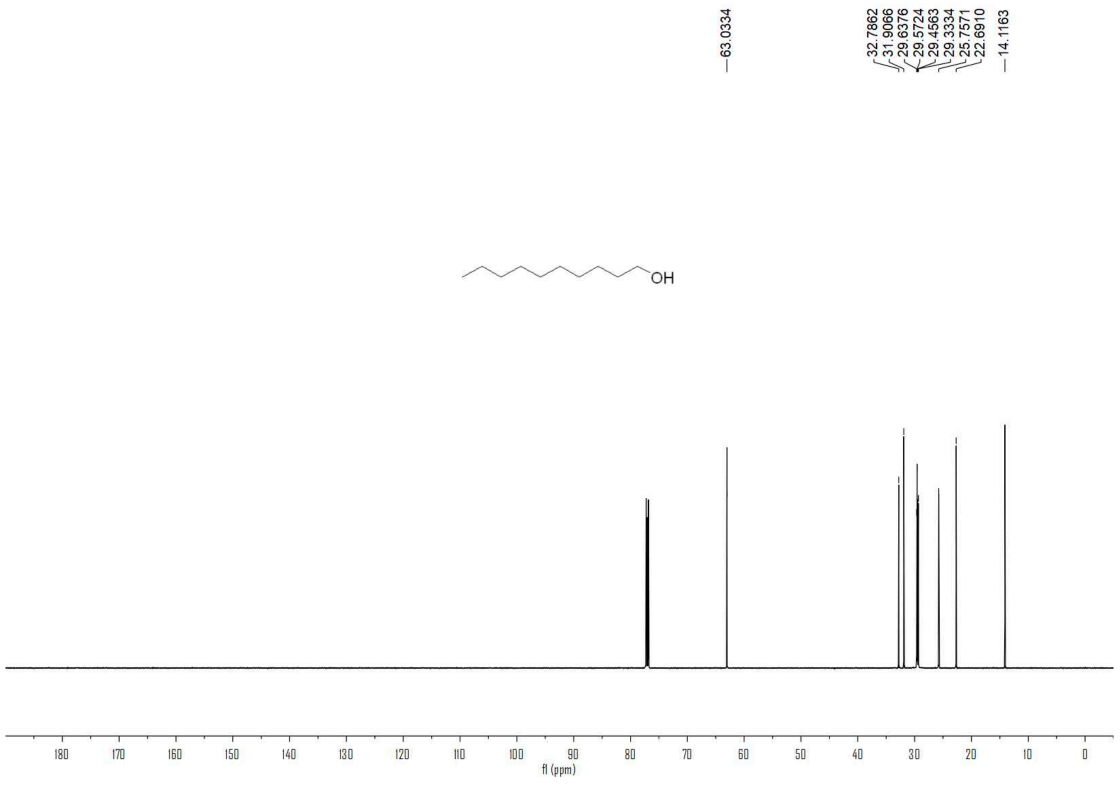

Supplement: Supplementary file 1 [file molecules-28-07915-s001.zip › molecules-2740911-supplementary.pdf]
